# Liquid biopsy reveals the immune status and protein profiles linked to CTC burden and clinical outcomes in metastatic breast cancer

**DOI:** 10.1186/s13046-026-03709-3

**Published:** 2026-04-18

**Authors:** Keerthi Kurma, Thomas Bardol, Caroline Mollevi, Zahra Eslami-S, Françoise Garima, Marie Alexandre, Angélique Bobrie, Gerald Lossaint, Blandine Massemin, Véronique D’Hondt, Séverine Guiu, Laure Cayrefourcq, William Jacot, Catherine Alix-Panabières

**Affiliations:** 1 Laboratory of Rare Circulating Human Cells-University Medical Center of Montpellier, Montpellier, France; 2 CREEC/CANECEV, MIVEGEC (CREES), Université de Montpellier, CNRS, IRD, Montpellier, France; 3 European Liquid Biopsy Society (ELBS), Hamburg, Germany; 4 Institute Desbrest of Epidemiology and Public Health, Univ Montpellier, INSERM, CHU Montpellier, Montpellier, France; 5 Department of Medical Oncology, Institut du Cancer de Montpellier, Montpellier University, Montpellier, France; 6 Institut de Recherche en Cancérologie de Montpellier, INSERM U1194, Montpellier University, Montpellier, France; 7 Department of Clinical Research and Innovation, Montpellier Cancer Institute (ICM), University of Montpellier, Montpellier, France; 8 Biological Resources Center, Montpellier Cancer Institute (ICM), University of Montpellier, Montpellier, France

**Keywords:** Circulating tumor cells, Circulating immune cells, Circulating proteins, Breast cancer, Progression-free survival, Overall survival

## Abstract

**Background:**

Metastatic breast cancer (mBC) remains a major therapeutic challenge. Increasing evidence suggests that metastatic dissemination and resistance to treatment are sustained by systemic immune dysfunction and vascular remodeling. Yet, the underpinning mechanisms remain incompletely understood. Therefore, we applied a multilayered liquid biopsy strategy to characterize the systemic immunovascular landscape of mBC and identify clinically actionable circulating biomarkers of disease progression and risk stratification.

**Methods:**

In this prospective study, peripheral blood samples were collected from 60 patients at diagnosis of stage IV mBC before treatment initiation. Circulating tumor cells (CTCs) were enumerated with the CellSearch^®^ system. Immune profiling was performed by flow cytometry, and plasma proteomic analysis using the Olink^®^ 96 Immuno-Oncology panel. Progression-free survival (PFS) was analyzed using Kaplan–Meier and Cox proportional hazards models, including multivariate analysis to identify independent prognostic factors.

**Results:**

Tumors were mainly HR⁺/HER2⁻ (83.3%), and the median follow-up and progression-free survival (PFS) were 31.5 months and 19.9 months, respectively. CTCs were detected in 58.3% of patients (range: 1–962 CTCs/7.5 mL blood). Comprehensive immune profiling revealed that total leukocytes and CD3⁺ T cells were strongly reduced and regulatory T cells (Tregs; CD4⁺CD25^high^CD127^low/–^), which expressed various inhibitory receptors (PD-1, CTLA-4, TIGIT, LAG-3), were increased in patient’s vs. age-matched healthy donors. The co-expression of PD-1, CTLA-4, LAG-3 and TIGIT in effector T cells (CD4⁺, CD8⁺) indicated exhaustion. Circulating monocytes/macrophages exhibited a shift toward the immunosuppressive CD163⁺CD206⁺ phenotype. Plasma proteomic profiling identified an inflammatory and angiogenic signature (elevated IL-6, IL-8, HGF, ANGPT2, NOS3, CSF1 and galectin-9, and reduced FASLG and TWEAK expression). Notably, Treg proportion and IL-8, HGF, galectin-9 and TNFRSF12A levels were higher in CTC-positive than CTC-negative patients. Liver metastases, triple-negative receptor status, and high CTC count predicted shorter PFS. Multivariate Cox analysis identified IL-8 (HR = 1.49, *p* = 0.004) and NOS3 (HR = 4.72, *p* = 0.003) as independent PFS predictors.

**Conclusion:**

This integrated cellular and proteomic liquid biopsy approach showed that mBC is a systemic immunovascular disease characterized by T-cell exhaustion, Treg expansion, TIGIT-mediated cytotoxic restraint, monocyte polarization, pro-angiogenic cytokine milieu and CTC-mediated immune escape. Circulating IL-8 and NOS3 may represent blood-based prognostic biomarkers and therapeutic targets in mBC.

**Trial registration:**

NCT04025541 registered on July 19, 2019.

**Supplementary Information:**

The online version contains supplementary material available at 10.1186/s13046-026-03709-3.

## One sentence summary

Liquid biopsy profiling reveals profound immune remodeling in metastatic breast cancer and highlights IL-8 and NOS3 as strong markers of disease progression.

## Introduction

Metastatic breast cancer (mBC) is the leading cause of cancer-related mortality in women worldwide [1, 2]. Despite advances in targeted, endocrine, and immunomodulatory drug treatments, mBC remains largely incurable, and the median overall survival (OS) rarely exceeds three to five years, depending on the subtype and treatment history [3]. Conventionally, mBC biology studies have focused on tumor-intrinsic mechanisms, such as receptor signaling, genomic instability and transcriptional plasticity. However, increasing evidence suggests that the metastatic process is determined not only by the tumor cell intrinsic aggressiveness but also by the host systemic environment [4–6]. The immune system and soluble inflammatory mediators jointly shape a pro-metastatic milieu that promotes tumor dissemination, immune escape and colonization of distant organs [7, 8]. Understanding this systemic reprogramming is essential to identify new therapeutic targets and refine the prognostic assessment in patients with advanced disease.

The immune system plays a dual role in cancer progression: it may constrain but also promote tumor development [9]. In early-stage breast cancer, effective immune surveillance has been associated with improved outcomes and better response to chemotherapy or immunotherapy [10, 11]. In advanced disease, the immune landscape often shifts toward chronic inflammation, exhaustion and regulatory dominance, ultimately favoring tumor progression [12, 13]. Previous studies showed that tumor-infiltrating lymphocytes and immune checkpoint molecule expression in the tumor microenvironment can influence the response to therapy, especially in triple-negative [14] or Human Epidermal Growth Factor Receptor 2-positive (HER2^+^) breast cancer [15]. However, far less is known about how peripheral blood component changes mirror the systemic immunity changes in mBC and how they relate to clinical outcomes [16]. As metastasis involves complex interactions between circulating tumor cells (CTCs), immune cells, and endothelial compartments, deciphering the circulating immune profile offers a unique opportunity to capture in real-time the systemic biology in mBC [17–20].

Liquid biopsy technologies provide an unprecedented non-invasive opportunity to uncover this multidimensional biology [21, 22]. Besides the detection of CTCs and circulating tumor DNA, liquid biopsy can be harnessed to study immune cell phenotypes, checkpoint molecule expression, and soluble cytokine or chemokine profiles [17, 23]. This approach allows monitoring in real time tumor-derived and host-derived factors that collectively determine the disease trajectory.

We hypothesized that in mBC, integrating immunophenotypic/proteomic data from peripheral blood samples with clinical features may yield valuable insights into the mechanisms driving disease progression and resistance to therapy. Therefore, the aim of the present study was to use a multilayered liquid biopsy approach that combines the analysis of cellular and soluble biomarkers to comprehensively characterize the systemic immune landscape in patients at diagnosis of mBC before metastatic disease treatment initiation [24]. By combining flow cytometry, proteomic profiling and clinical data, we sought to understand how immune cell composition, checkpoint molecule activation, monocyte/macrophage polarization and soluble mediator expression interact to shape the metastatic state, and to evaluate how this integrated profiling strategy could translate into clinically relevant risk stratification. We also wanted to determine whether specific circulating molecules could serve as prognostic biomarkers of disease progression with potential applicability for patient monitoring and therapeutic decision-making.

Through this integrated liquid biopsy-based approach, we found that mBC is not simply a manifestation of tumor cell dissemination, but a systemic disorder characterized by coordinated immune cell reprogramming. We identified widespread T-cell exhaustion, expansion of regulatory T cells (Treg) that express different inhibitory receptors, natural killer (NK) cell functional restraint through TIGIT signaling, and monocyte polarization toward the immunosuppressive CD163⁺CD206⁺ phenotype. Moreover, we uncovered an inflammatory and pro-angiogenic proteomic signature dominated by secreted IL-8 and NOS3, which emerge as two independent predictors of progression-free survival (PFS). These findings redefine mBC as a systemic immunovascular disease and highlight IL-8 and NOS3 as clinically accessible biomarkers of mBC aggressiveness.

## Materials and methods

### Study design

A total of 60 patients (≥ 18 years) with histologically confirmed stage IV breast cancer were prospectively included in the ALCINA 2 basket clinical trial (NCT04025541). Recruitment took place at the Montpellier Cancer Institute (ICM) between April 2022 and April 2023, prior to initiation of first-line metastatic treatment. Patients with history of previous malignancies (excepted in situ cervix cancer and non-invasive skin cancer) and pregnant women were excluded. Clinical baseline characteristics were retrieved from electronic health records in accordance with the Biospecimen Reporting for Improved Study Quality (BRISQ) guidelines, as already published [24]. For comparison, 21 age-matched healthy female donors were recruited through the Établissement Français du Sang (EFS). Eligibility of healthy donors was assessed using the standard EFS blood donor criteria, including age ≥ 18 and < 70 years and body weight > 50 kg, after a pre-donation medical interview. Donors had no history of malignancy, no ongoing acute infection or inflammatory disease, no recent vaccination (< 4 weeks), and no recent surgical or endoscopic procedure (< 4 weeks). Additional exclusion criteria included history of sexually transmitted diseases or high-risk behaviors, recent tattoos or piercings (< 2 months), and use of intravenous or intramuscular substances.

At inclusion, patient blood samples were collected in 10 mL CellSave^®^ tubes (Menarini Silicon Biosystems, ref: 7900005) and EDTA tubes by the Biological Resource Center of the Montpellier Cancer Institute (CRB-ICM, BB-0033-00059 (Fig S1)). Tubes were stored at room temperature and transferred to the Laboratory of Rare Human Circulating Cells and Liquid Biopsy (LCCRH), Montpellier University Hospital, for processing within 24 h (EDTA tubes) or 72 h (CellSave^®^ tubes). HD samples were collected in 6 mL purple-cap BD Vacutainers^®^ containing EDTA (BD-Plymouth ref: 367864 A) and processed at the University Medical Center of Montpellier. This study was approved by a national ethics committee (Comité des Personnes Ouest IV Nantes, accepted on March 7, 2018) and by the French National Agency for the Safety of Medicines and Health Products (reference ID-RCB: 2018-A00064-51, accepted on March 8, 2018), and written informed consent was obtained from all patients and HDs.

### Patient data collection

Patients were included during 12 consecutive months after discussion in a multidisciplinary team meeting that included at least, a radiologist, an oncologist, and a general surgeon to discuss treatment options. Patients’ data were prospectively collected (Table 1).

**Table 1:** Overview of clinical characteristics, treatment history, and outcomes of patients with mBC

Patient data	
Medical history	Past personal and family history relevant to the pathology
Initial cancer diagnosis	Including the date and status at diagnosis
Cancer history	Detailed medical history related to the malignant disease
Lifestyle factors	Including smoking status, alcohol consumption, physical activity, and other relevant habits
Clinical imaging results	Radiological assessments performed during the disease course
Laboratory test results	Routine laboratory findings and relevant biomarkers
Histopathological findings	Pre- and/or post-treatment histological and pathological assessments
Clinical documentation	Medical reports (hospitalization and consultation reports)
Chemotherapy and treatment response	Type and regimen; assessment of the clinical and morphological response based on the RECIST guidelines
Disease progression	Date and type of progression, based on clinical and imaging criteria
Survival data	Date and cause of death, if applicable

Patients’ follow-up was terminated at new disease progression, death or at the end of the defined 3-year follow-up period in case of uneventful disease course, as described in the protocol (ClinicalTrials.gov NCT04025541).

### CTC detection

CTCs were identified using the FDA-cleared CellSearch^®^ system (Menarini Silicon Biosystems, Inc., Bologna, Italy). Blood samples were processed with the CellSearch^®^ CXC kit (ref: 7900017) and the CellTracks^®^ Autoprep^®^ System, following the manufacturer’s protocols. Briefly, 7.5 mL of blood was used to isolate and capture EpCAM-positive CTCs through a positive-enrichment method. Detection involved staining CTCs with fluorescein isothiocyanate-conjugated anti-cytokeratin antibodies (CK-8, -18, and − 19) and anti-CD45 allophycocyanin antibodies to exclude leukocytes. Nuclear staining was performed with DAPI. Additionally, a user-defined PE-conjugated antibody (targeting AXL in this study) was incorporated *via* the fourth detection channel as previously described in our proof-of-concept study in the same patient cohort [24]. Following immunocytochemical staining, immunomagnetically labeled cells were retained in a magnetic field and analyzed using the CellTracks Analyzer II^®^. All detected events were reviewed by a certified technician, and an experienced biologist based on standardized criteria to confirm CTC isolation. Events were classified as CTCs according to established CellSearch^®^ criteria, including presence of a DAPI-positive nucleus, cytokeratin positivity, CD45 negativity, appropriate cellular size and morphology, intact cellular outline, and absence of apoptotic debris. All candidate events underwent dual review, and in case of ambiguity, consensus was reached to minimize inter-reader variability. Given the exploratory nature of the study, a threshold of 1 CTC per 7.5 mL of blood was set to classify a sample as CTC-positive (CTC^+^).

### Peripheral Blood Mononuclear Cell (PBMC) isolation and flow cytometry analysis

In this ancillary part of the study, PBMCs were isolated from whole blood samples (patients with mBC and HDs) using Ficoll-Paque™ (density: 1.077 g/mL) density gradient centrifugation. Blood collected in EDTA tubes was diluted 1:1 with sterile PBS and layered over the same volume of Ficoll (1:1 ratio). Samples were centrifuged at 1200 g at room temperature for 20 min, with the brake disabled to maintain gradient integrity. Following centrifugation, the PBMC layer was carefully aspirated using a sterile pipette, avoiding disturbance of the Ficoll layer. Isolated PBMCs were cryopreserved in liquid nitrogen using a freezing medium consisting of 90% fetal bovine serum and 10% DMSO. For multi-parametric flow cytometry analysis, cells were stained in a 96-well plate. Each well was loaded with 100 µL of cell suspension (1–2 × 10⁵ cells) and incubated with the antibody cocktail 1 [anti-CD45 (cloneHI30, BioLegend, AB_314402), anti-CD3 (clone UCHT1, BioLegend, AB_2562046), anti-CD8 (clone SK1, BioLegend, AB_2876774), anti-CD4 (clone-SK3, BioLegend, AB_2820224), anti-CD25 (clone BC96, BioLegend, AB_314278), anti-CD127 (clone A019D5, BioLegend, AB_10720185), anti-CD56 (Clone 5.1H11, BioLegend, AB_2566061), anti-CD19 (clone SJ25C1, BioLegend, AB_2616936), anti-CD279 (clone-EH12.2H7, BioLegend, AB_2562256), anti-CD152 (clone-BNI3, BioLegend, AB_2810582), anti-CD223 (clone-1C3C65, BioLegend, AB_2734422), anti-TIGIT (clone-741182, BD Biosciences, AB_2872303) antibodies] for the assessment of all T cells, cytotoxic T cells, T-regulatory cells (Tregs), natural killer (NK) cells, B cells, and checkpoint inhibitors or stimulatory checkpoint molecules. A second antibody cocktail [Cocktail 2: anti-CD45 (cloneHI30, BioLegend, AB_314402), anti-CD3 (clone UCHT1, BioLegend, AB_2562046), anti-CD19 (clone-SJ25C1, BioLegend, AB_2616936), anti-CD14 (clone-M5E2, BioLegend, AB_2563629), anti-CD11b (clone-ICRF44, BioLegend, AB_2632619), anti-CD80 (clone-W17149D, BioLegend, AB_2890820), anti-CD86 (clone-BU63, BioLegend, AB_2728392), anti-CD163 (clone-GHI/61, BioLegend, AB_2562463), anti-CD206 (clone-15-2, BioLegend, AB_571919) antibodies] was used to determine the expression profiles of monocytes (M1 and M2). Cells were incubated with the antibody cocktails at 4 °C in the dark for 30 min. Then, cells were washed with flow cytometry buffer, dead cells were excluded by staining with the Viakrome 808 viability dye (Beckman Coulter, Reference: C36628), and samples were analyzed on a CytoFLEX flow cytometer. All procedures were performed in sterile conditions, with appropriate controls included for accurate data interpretation.

### Plasma proteomic profiling using the Olink^®^ 96 Immuno-Oncology panel

Peripheral blood samples were collected from patients with mBC (before treatment initiation) and HDs into EDTA tubes. Plasma was isolated by centrifugation at 16,000 g for 20 min, aliquoted to avoid repeated freeze–thaw cycles, and stored at − 80 °C until analysis. Proteomic profiling was performed using the Olink^®^ Target 96 Immuno-Oncology panel (Olink Proteomics, Uppsala, Sweden) that simultaneously quantifies 92 proteins related to immune regulation, inflammation, angiogenesis, and tumor biology. The assay is based on the Proximity Extension Assay (PEA) technology, in which paired oligonucleotide-labeled antibodies bind to target proteins; subsequent hybridization and DNA polymerization generate a unique reporter sequence proportional to the protein concentration. Detection and quantification were carried out using a high-throughput microfluidic real-time PCR platform (Fluidigm Biomark™ HD system). Data were processed using the Olink standard workflow and expressed as Normalized Protein Expression units on a log2 scale. Inter-plate variation was corrected by internal and inter-plate controls included in each assay. Proteins with values below the assay-specific limit of detection (LOD) were excluded from downstream analysis.

### Statistical analysis

Statistical analyses were performed using the R software, version 4.4.0. Quantitative variables were summarized as median (range) and compared using the Wilcoxon–Mann–Whitney test. Qualitative variables were presented as numbers and percentages and compared using the Pearson’s Chi-square test or Fisher’s exact test when the expected frequency was < 5. Given the large number of markers assessed, q-values derived from false discovery rate (FDR) correction were calculated to account for multiple testing.

The median follow-up was estimated using the Schemper method. Survival curves were generated with the Kaplan–Meier method. Cox proportional hazard models incorporating selected clinical and biological covariates were used. Variables with *p* < 0.10 in univariate analysis and clinically relevant factors were included in the multivariate model, and backward covariate selection was applied. Hazard ratios (HR) were reported with their 95% confidence interval.

## Results

### Patient characteristics, breast cancer molecular subtypes, first-line treatments, metastatic site distribution and CTC detection define mBC heterogeneity

**Fig. 1:**
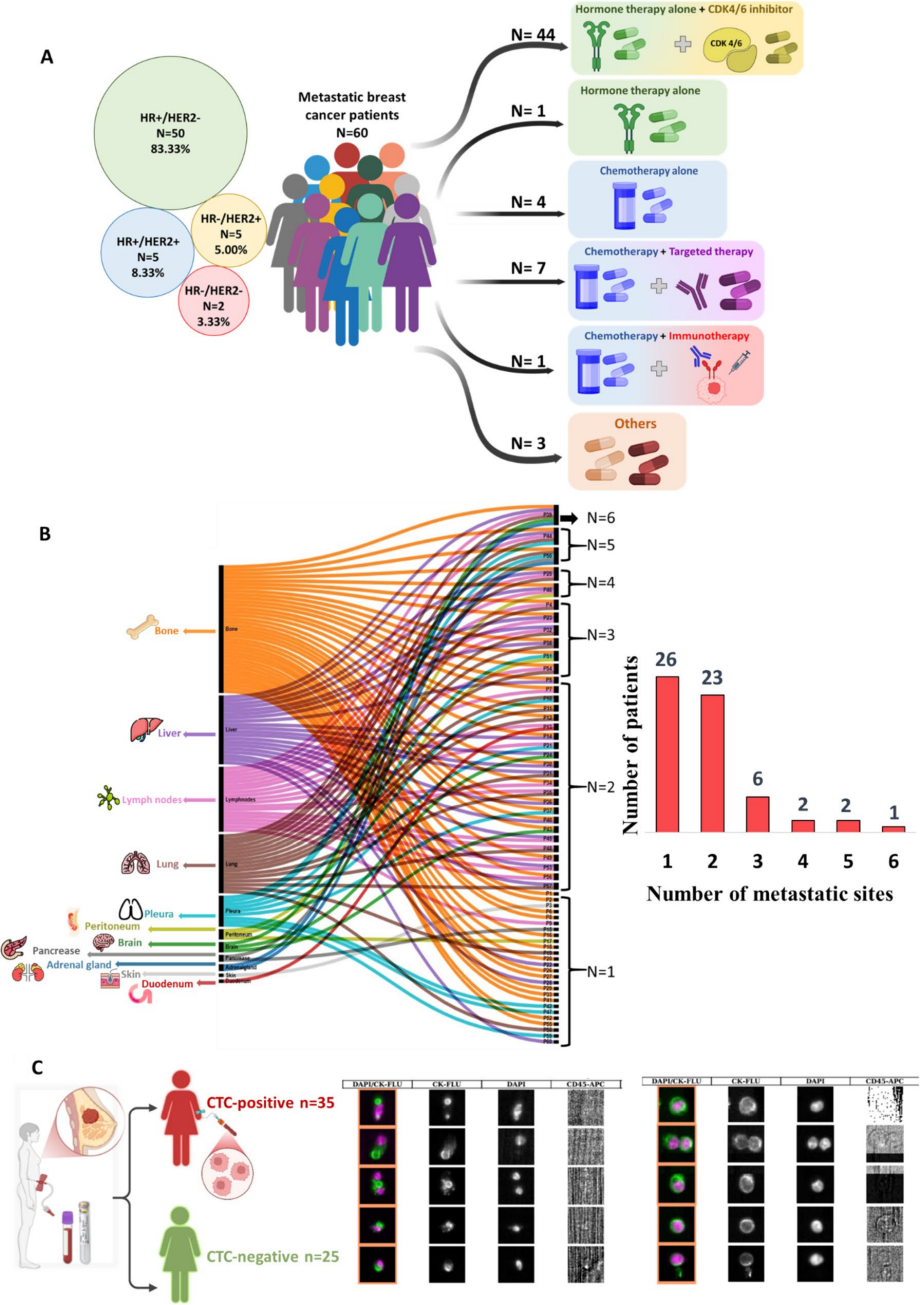
Clinical characteristics and CTC detection in patients with mBC.** A** Summary of the patients’ characteristics. Their mean age was 61.55 years (± 13.36) and most patients had HR^+^/HER2^−^ (83.3%, *n* = 50), followed by HR^+^/HER2^+^ (8.3%, *n* = 5), HR^−^/HER2^+^ (5.0%, *n* = 3), and triple-negative HR^−^/HER2^−^ cancer (3.3%, *n* = 2). First-line treatments included hormone therapy combined with CDK4/6 inhibitors, hormone therapy alone, chemotherapy alone, chemotherapy combined with targeted therapy, chemotherapy combined with immunotherapy, and other regimens. **B** Sankey diagram (left) showing the distribution of metastatic sites in the study cohort. The most frequent sites were bone, liver, lymph nodes and lung, followed by pleura, peritoneum, brain, pancreas, adrenal gland, skin and duodenum. Each line represents an individual patient. Histogram (right) showing the number of metastatic sites per patient. Most patients had one (*n* = 26) or two (*n* = 23) metastatic sites. **C** Schematic representation of CTC analysis in the 60 patients with mBC using the CellSearch^®^ platform: 35 were classified as CTC-positive (≥ 5 CTCs/7.5 mL of blood), and 25 as CTC-negative. Representative images of CTCs identified by CellSearch^®^. CTCs were defined as EpCAM^+^ (based on the enrichment step), nucleated (DAPI⁺), cytokeratin-positive (CK⁺/FITC), and CD45⁻ (exclusion marker, APC) cells. Images show merged and individual fluorescence channels for both CTC-positive and CTC-negative samples

We collected peripheral blood samples from 60 patients with stage IV mBC before first-line treatment for metastatic disease (Fig. S1A). The median age at inclusion was 63.5 years [33.00; 89.00]. The predominant histological subtype was ductal invasive (no special type) carcinoma (50/60 patients; 83%). Moreover, 83% (50/60) of patients had a hormone receptor (HR)^+^/HER2^−^ tumor, 8% (5/60) a HR^+^/HER2^+^ tumor, 5.0% (3/60) a HR^−^/HER2^+^ tumor, and 3% (2/60) a triple-negative (HR^−^/HER2^−^) tumor (Fig. 1A). For the metastasis management, 73% (44/60) of patients received hormone therapy with CDK4/6 inhibitors, 1.7% (1/60) only hormone therapy, 6.7% (4/60) chemotherapy alone, 11% (7/60) chemotherapy with targeted therapy, and 1.7% (1/60) chemotherapy with immunotherapy (Fig. 1A). The median time from the initial breast cancer diagnosis to study inclusion (i.e. metastasis detection) was 40 months (range: 0–366 months). Thirty-seven patients (62%) presented metachronous metastases (detection > 6 months after the primary tumor diagnosis), with a median time from diagnosis to metastatic disease of 44 months.

The mean number of metastatic sites was 2 [1–6]. Metastatic disease was detected at a single site in 26/60 patients, at two sites in 23 patients, at three sites in 6 patients, at four sites in 2 patients, five sites in 2 patients, and at six sites in 1 patient. Bone was the most frequent metastasis site (*n* = 37, 62%), followed by liver (*n* = 20, 33%), lung (*n* = 17, 28%) and lymph nodes (*n* = 15, 25%) (Fig. 1B). Notably, 34/60 patients had metastases in multiple organs, particularly in bone and lymph nodes (*n* = 10/34; 29%).

Using the CellSearch^®^ system, we identified at least 1 CTC in the blood sample of 35/60 patients (58.3%). CTC numbers ranged from 1 to 962 (Fig. 1C, Table S1).

### T-cell exhaustion and Treg expansion underpin systemic immune suppression in mBC

**Fig. 2:**
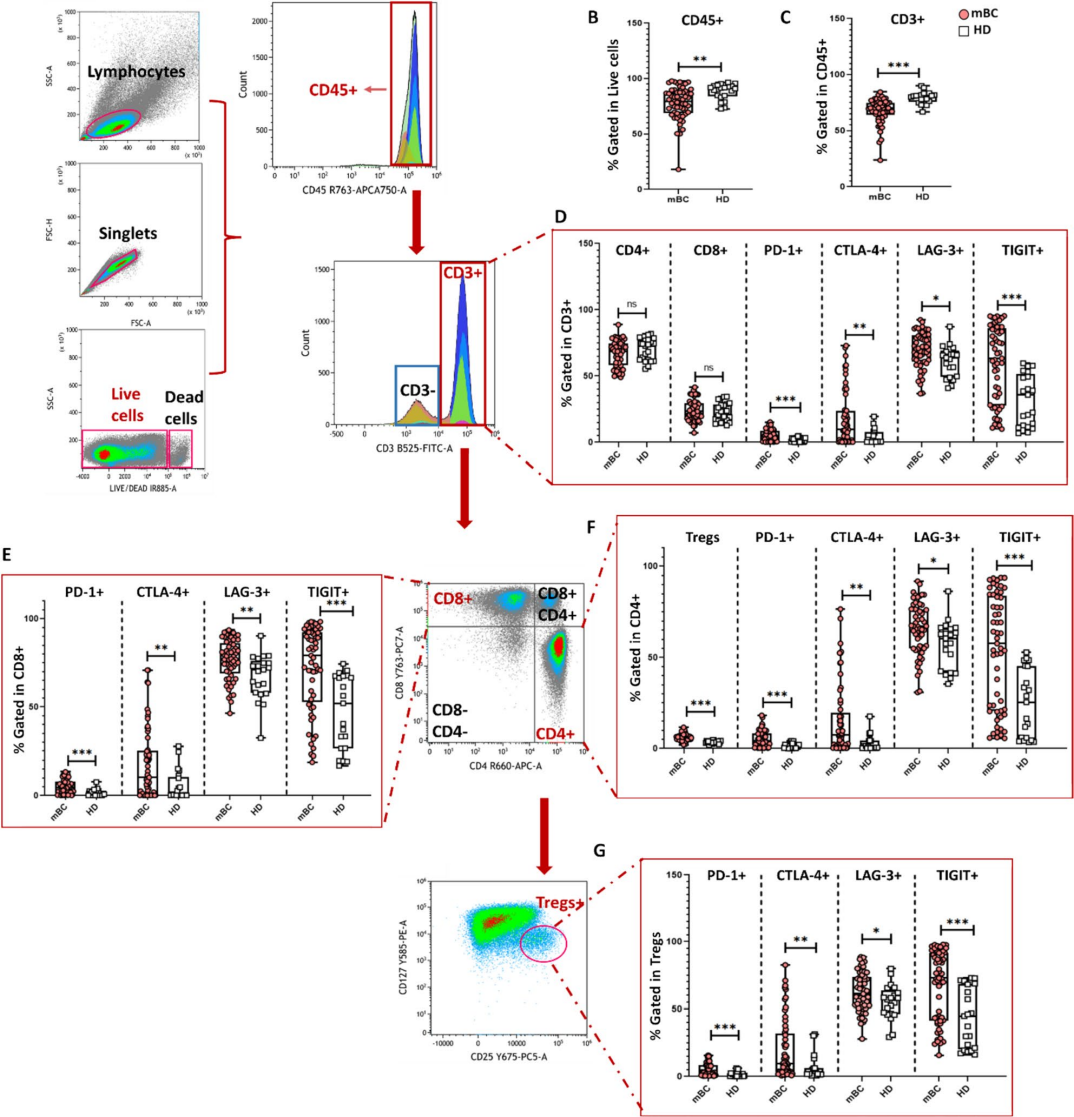
Comparative analysis of T-cell subsets and immune checkpoints in patients with mBC and HDs. **A** PBMCs were isolated and analyzed by multiparametric flow cytometry. Sequential gating was applied to identify lymphocytes, singlets and live cells, followed by selection of CD45⁺ and CD3⁺ T cells. Within the CD3⁺ compartment, the CD4⁺ and CD8⁺ subsets and Tregs (CD4⁺CD25⁺CD127^low/−^) were identified. **B-C** Box and violin plots showing the proportion (mean ± SEM) of **(B)** CD45⁺ cells and **(C)** of CD3⁺ T cells (within the CD45⁺ compartment) in patients with mBC and HDs. **D** Box and violin plots showing the proportions (mean ± SEM) of CD8⁺ and CD4⁺ subsets (within the CD3⁺ compartment) and of CD3⁺ cells that express PD-1, CTLA-4, LAG-3, and TIGIT in patients with mBC and HDs. **E–F** Box and violin plots showing the proportions (mean ± SEM) of CD8⁺ (E) and CD4⁺ (F) cells that express PD-1, CTLA-4, and TIGIT in patients with mBC and HDs. **G** Box and violin plots showing the proportions (mean ± SEM) of Tregs that express PD-1, CTLA-4, LAG-3 and TIGIT in patients with mBC and HDs. *p < 0.05, **p < 0.01, ***p < 0.001, ****p < 0.0001; ns, not significant (nonparametric Wilcoxon–Mann–Whitney test)

We then assessed the composition of circulating T-cell populations in patients with mBC and age-matched HDs by flow cytometry. We used the gating strategy described in Fig. 2 to ensure robust identification of T-cell subsets. After selecting lymphocytes based on their FSC/SSC properties, we excluded doublets and dead cells and gated CD45⁺ leukocytes. The proportion of CD45⁺ cells was significantly lower in patients with mBC than HDs (*p* = 0.001, Fig. 2B, Table S2). Within this population, we identified CD3⁺ T cells and subdivided them into the CD4⁺ and CD8⁺ subsets. Again, the proportion of CD3⁺ T cells (as a percentage of all CD45⁺ cells) was significantly lower in patients with mBC (*p* < 0.001, Fig. 2C, Table S2), reflecting broad impairment of circulating T cells. Conversely, the proportions of CD4⁺ and CD8⁺ T cells (as a percentage of all CD3⁺) were similar between patients with mBC and HDs (Fig. 2D).

To investigate the T-cell subset functional state, we analyzed the relative frequency of key immune checkpoint molecules (PD-1, CTLA-4, LAG-3, and TIGIT), which act as inhibitory receptors to regulate T-cell activation and maintain immune balance, in CD3⁺ T cells and the CD4⁺ and CD8⁺ subsets. The percentages of gated CD3⁺ PD-1⁺ T cells (*p* < 0.001, Fig. 2D, Table S2) and of CD8⁺ PD-1⁺ and CD4⁺ PD-1⁺ T cells (*p* < 0.001 for both, Fig. 2E and F, Table S2) were higher in patients with mBC than HDs. Similarly, the percentages of CTLA-4⁺ T cells (CD3⁺, *p* = 0.002, Fig. 2D; CD8⁺, *p* = 0.001, Fig. 2E; and CD4⁺, *p* = 0.002, and Fig. 2F, Table S2), LAG-3⁺ T cells ( CD3⁺, *p* = 0.013, Fig. 2D; and CD8⁺, *p* = 0.004, Fig. 2E; and CD4⁺, *p* = 0.013, Fig. 2F, Table S2), and TIGIT⁺ T cells (CD3⁺; CD8⁺; CD4⁺; *p* < 0.001, Fig. 2D, E and F, Table S2) were higher in patients with mBC than HDs.

Moreover, the proportion of Tregs (CD4⁺CD25^high^CD127^low/−^) was increased in patients with mBC compared with HDs (*p* < 0.001, Fig. 2F, Table S2) as well the proportions of Tregs that expressed PD-1, CTLA-4 or TIGIT (*p* < 0.001, *p* = 0.003 and *p* > 0.001, Table S2) (Fig. 2G). We observed a similar trend for LAG-3⁺ expression, although the difference was not significant (*p* = 0.058, Fig. 2G, Table S2).

These results reveal a clear pattern of systemic immune dysregulation in mBC. Circulating immune cell populations were markedly reduced (total leukocytes and T cells), consistent with an overall weakening of the immune surveillance in advanced disease. The higher proportions of CD3⁺, CD4⁺ and CD8⁺ cells that expressed immune checkpoint molecules (PD-1, CTLA-4, LAG-3, and TIGIT) indicated a widespread activation of inhibitory signaling pathways that contribute to T-cell exhaustion and functional impairment. In parallel, the expansion of Treg cells that express inhibitory receptors highlights an additional layer of immunosuppression, reinforcing the dominance of regulatory mechanisms over effector immunity in mBC.

### The expanded but inhibited NK cell compartment and B-cell depletion underscore the immune suppression in mBC

**Fig. 3:**
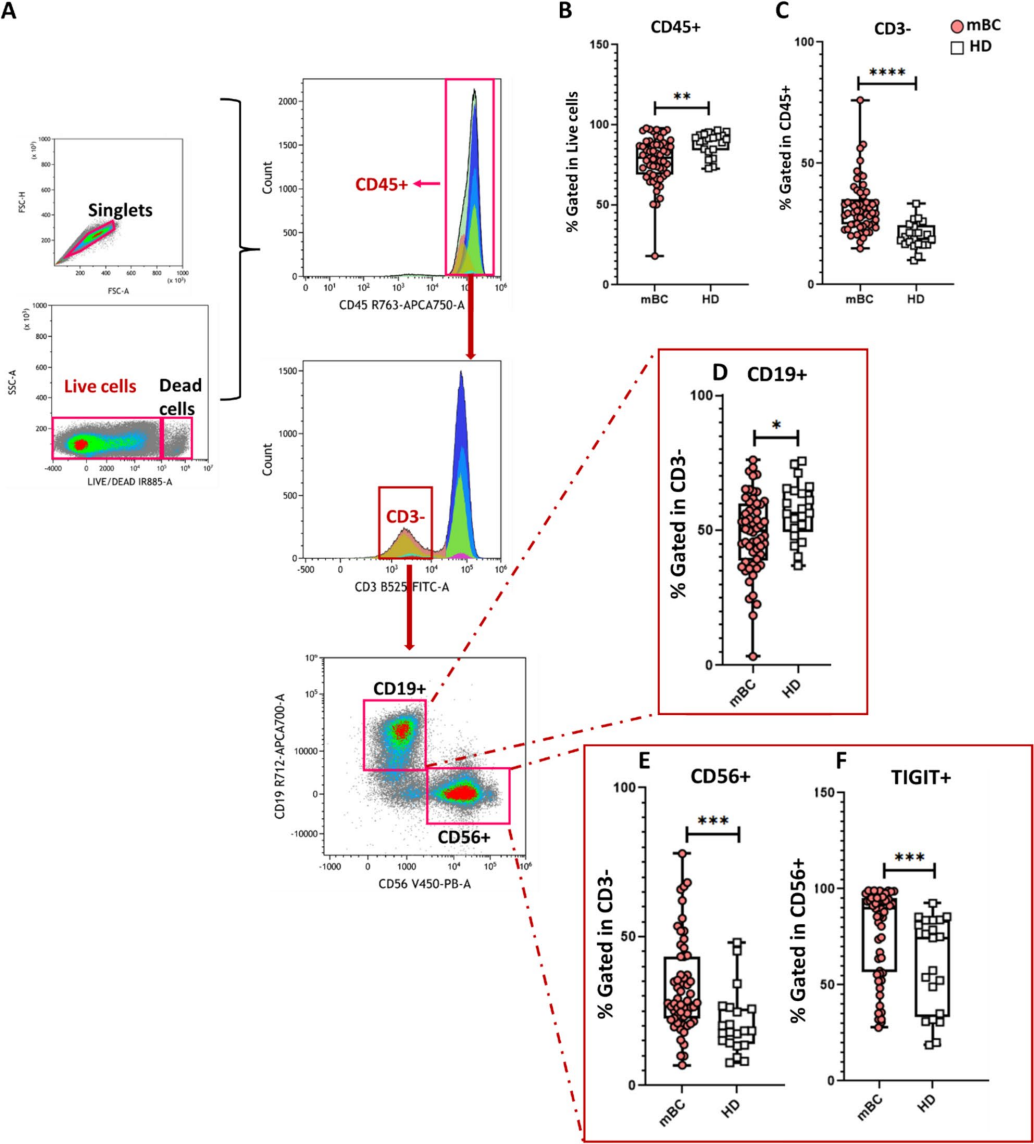
Comparative analysis of circulating B and NK cells in patients with mBC and HDs. **A** PBMCs were analyzed by multiparametric flow cytometry. Sequential gating identified lymphocytes, singlets and live cells, followed by selection of CD45⁺ cells. Within this population, CD3⁻ cells were gated to exclude T cells and to define non-T-cell subsets. Among all CD3⁻ cells, CD19⁺ B cells and CD56⁺ NK cells were selected. **B-C** Box and violin plots showing the proportion (mean ± SEM) of CD45^+^ (B) and CD3⁻ (C) cells in patients with mBC and HDs. **D** Box and violin plots showing the proportion (mean ± SEM) of CD19⁺ B cells among all CD3⁻ cells in patients with mBC and HDs. **E-F** Box and violin plots showing the proportions (mean ± SEM) of CD56⁺ NK cells (E) and of NK cells that express the inhibitory receptor TIGIT in patients with mBC and HDs. *p < 0.05, **p < 0.01, ***p < 0.001, ****p < 0.0001; ns, not significant (nonparametric Wilcoxon–Mann–Whitney test)

Flow cytometry analysis of circulating CD3^−^ cells revealed significant differences between patients with mBC and HDs (gating strategy in Fig. 3A-C). The proportion of circulating CD19⁺ cells (B cells, as a percentage of all CD3^−^ cells) was decreased in patients with mBC (*p* = 0.018, Fig. 3D, Table S2). Conversely, the proportion of NK cells (CD56⁺ cells) was higher in patients with mBC than HDs (*p* < 0.001, Fig. 3E, Table S2). However, the proportion of CD56⁺TIGIT⁺ NK cells also was increased in patients with mBC compared with HDs (*p* < 0.001, Fig. 3F, Table S2). As TIGIT is an inhibitory receptor that limits NK cell cytotoxicity, this finding indicates a state of functional exhaustion or immune suppression despite numerical expansion. It also suggests a complex remodeling of the NK cell compartment, where an enhanced cytotoxic potential may coexist with effector function suppression.

Collectively, these findings reveal a disrupted immune balance in mBC, characterized by a decreased proportion of B cells, expanded but functionally restrained NK cells, and a shift toward immune suppression. These alterations might contribute to systemic immune dysregulation and tumor immune escape mechanisms.

### The systemic increase of CD163⁺CD206⁺ monocytes reveal the immunosuppressive reprogramming in mBC

**Fig. 4:**
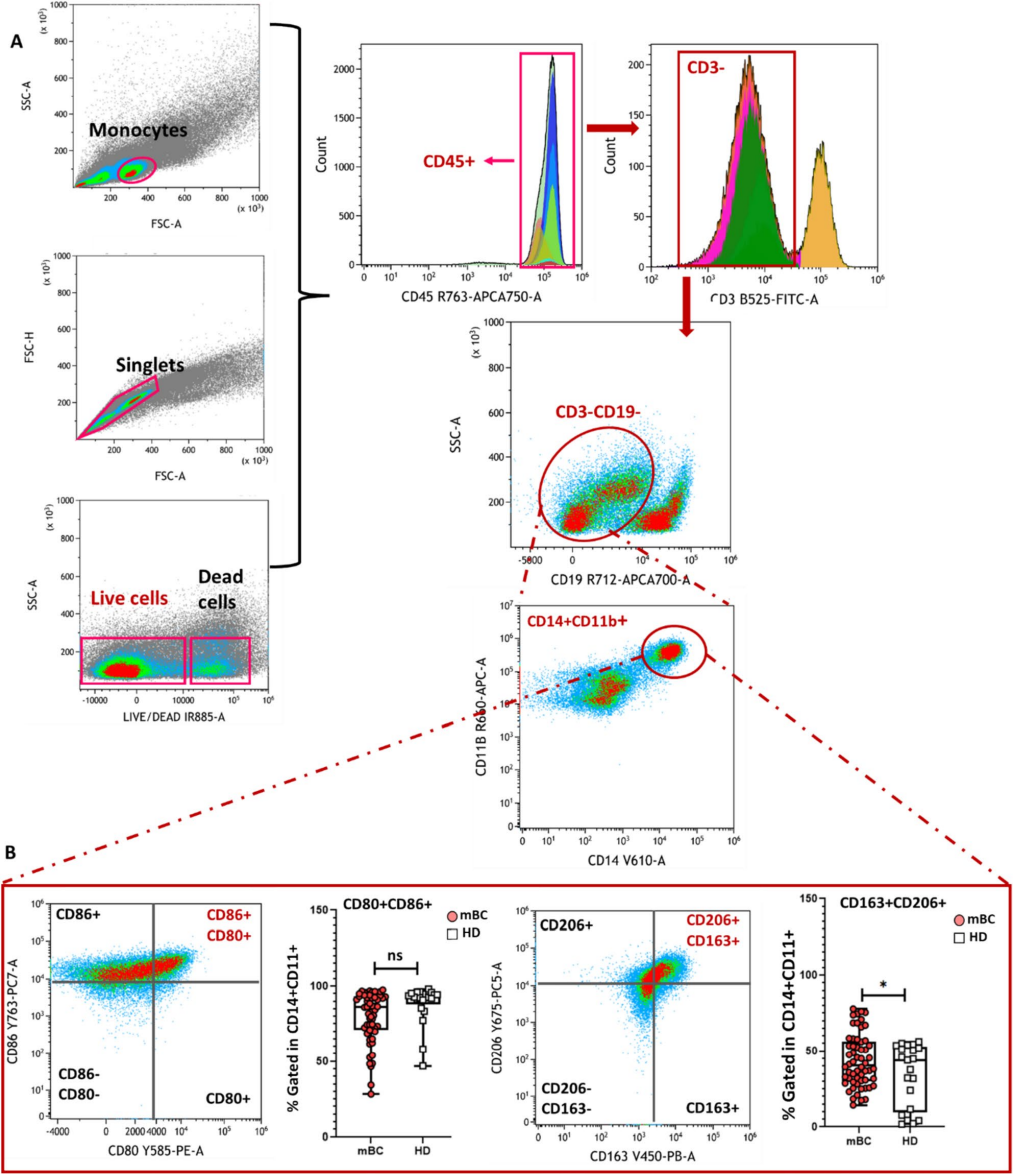
Comparative analysis of circulating monocyte subsets in patients with mBC and HDs. **A** PBMCs were analyzed by multiparametric flow cytometry. Monocytes were identified based on their FSC/SSC properties, followed by exclusion of doublets and dead cells. CD45⁺ leukocytes were gated, and CD3⁻CD19⁻CD14⁺CD11b⁺ cells were selected to define the monocyte/macrophage population. Within this population, CD80⁺CD86⁺ cells were identified as classically activated (M1-like) monocytes and CD163⁺CD206⁺ cells as alternatively activated (M2-like) monocytes. **B** Box and violin plots showing the proportions (mean ± SEM) of CD80⁺CD86⁺ monocytes/macrophages (left) and of CD163⁺CD206⁺ monocytes/macrophages (right) in patients with mBC and HDs. *p < 0.05; ns, not significant (nonparametric Wilcoxon–Mann–Whitney test)

To characterize the circulating monocyte subsets, we first identified monocytes based on their forward and side scatter properties, followed by exclusion of doublets and dead cells using FSC-H/FSC-A and LIVE/DEAD discrimination, respectively (Fig. 4A). Briefly, we gated CD45⁺ leukocytes, and then excluded T cells (CD3⁺) and B cells (CD19⁺) to enrich for myeloid cells. Within the CD3⁻CD19⁻ fraction, we selected CD14⁺CD11b⁺ cells to define the monocyte population: classically activated monocytes (M1-like, pro-inflammatory; CD80⁺CD86⁺) and alternatively activated monocytes (M2-like, anti-inflammatory; CD163⁺CD206⁺).

The proportion of CD163⁺CD206⁺ monocytes (percentage of CD3^−^CD19^−^CD14⁺CD11b⁺ cells) was higher in patients with mBC than HDs (*p* < 0.001, Fig. 4B, Table S2). As CD163 and CD206 are associated with tissue repair, immune suppression and tumor progression, these circulating immunosuppressive M2-like monocytes may contribute to a systemic environment conducive to metastasis and immune evasion in mBC. Conversely, the proportion of CD80⁺CD86⁺ monocytes (percentage of CD3⁻CD19⁻CD14⁺CD11b⁺ cells), which are associated with antigen presentation and T-cell co-stimulation, tended to be lower in patients with mBC (*p* = 0.172, Fig. 4B, Table S2).

Overall, these findings reveal a profound remodeling of the circulating monocyte/macrophage compartment in mBC, characterized by a shift from pro-inflammatory, immune-stimulatory subsets toward alternatively activated, immunosuppressive populations.

### Plasma proteomic profiling reveals systemic inflammatory, angiogenic and immunoregulatory remodeling in mBC

To describe the systemic immune alterations associated with mBC, we profiled plasma samples from patients with mBC and HDs using a 96-protein immuno-oncology panel that includes cytokines, chemokines, growth factors, immune checkpoints and tumor-associated proteins. Comparison of the obtained profiles revealed a distinct immune signature in mBC, characterized by inflammatory, angiogenic and immunoregulatory shifts.

**Fig. 5:**
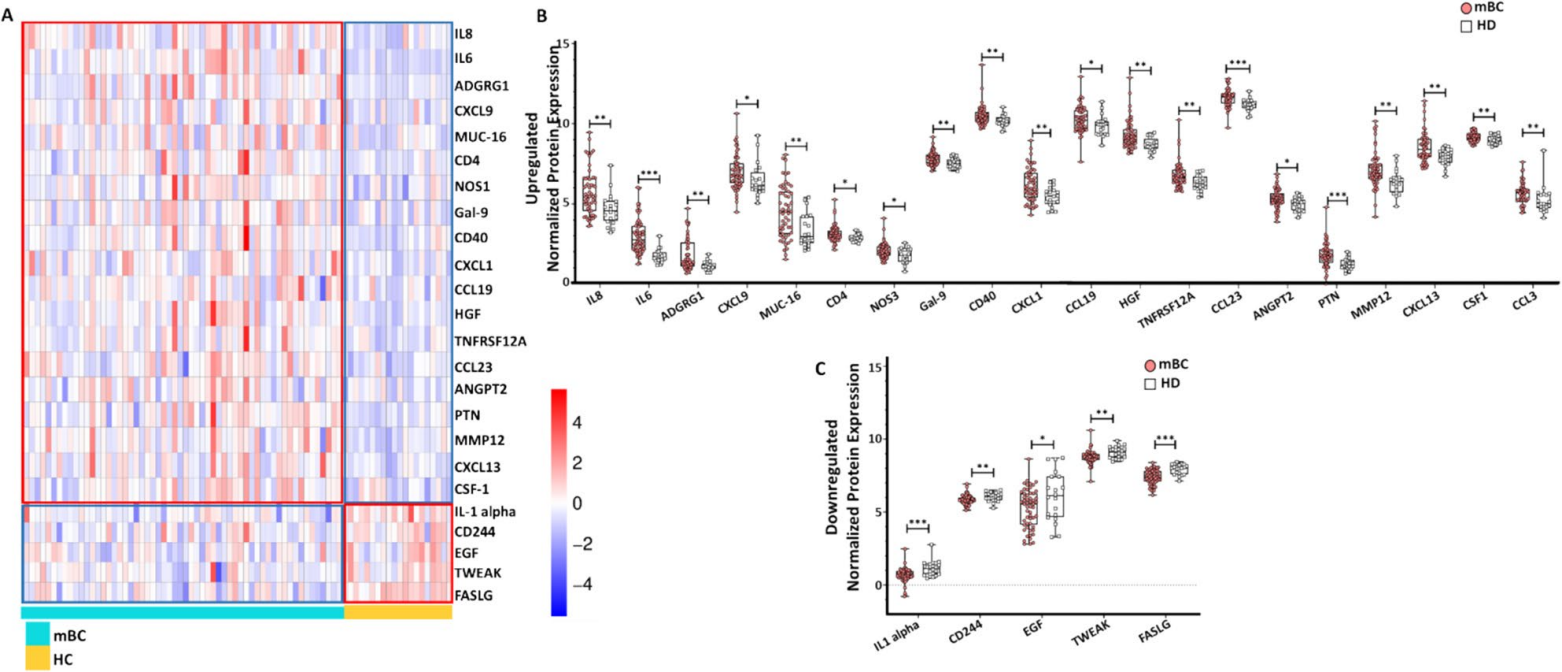
Different plasma protein profiles in patients with mBC and HDs. **A** Heatmap showing the expression patterns of differentially expressed plasma proteins across individual samples. Unsupervised clustering separated the mBC and HD groups. **B-C** Box and violin plots illustrate the distribution of upregulated (B) and downregulated (C) proteins in patients with mBC compared with HDs. *p < 0.05, **p < 0.01, ***p < 0.001; ns, not significant (nonparametric Wilcoxon–Mann–Whitney test)

Specifically, 20 of the 92 proteins analyzed were significantly upregulated and 5 downregulated in patients with mBC (Fig. 5). Proteins upregulated (higher normalized protein expression) in mBC compared with HD plasma samples included pro-inflammatory cytokines, such as interleukin-6 (IL-6) and interleukin-8 (IL-8); the chemokines C-X-C motif chemokine ligand 9 (CXCL9), C-X-C motif chemokine ligand 1 (CXCL1), C-X-C motif chemokine ligand 13 (CXCL13), C-C motif chemokine ligand 19 (CCL19), C-C motif chemokine ligand 23 (CCL23), and C-C motif chemokine ligand 3 (CCL3); the angiogenesis and growth markers hepatocyte growth factor (HGF), angiopoietin-2 (ANGPT2), pleiotrophin (PTN), and nitric oxide synthase 3 (NOS3); the immune-related markers cluster of differentiation 40 (CD40), cluster of differentiation 4 (CD4) and galectin-9 (LGALS9); the tumor-associated proteins mucin-16 (MUC16) and adhesion G protein-coupled receptor G1 (ADGRG1); the matrix-related matrix metalloproteinase-12 (MMP12); and the myeloid-associated proteins colony-stimulating factor 1 (CSF1) and tumor necrosis factor receptor superfamily member 12 A (TNFRSF12A/Fn14) (Fig. 5B).

Conversely, interleukin-1 alpha (IL1A), cluster of differentiation 244 (CD244), epidermal growth factor (EGF), tumor necrosis factor ligand superfamily member 12 (TWEAK/TNFSF12), and Fas ligand (FASLG) were significantly downregulated in mBC samples compared with HDs (Fig. 5C).

Overall, these results define a distinct systemic proteomic signature of mBC, characterized by a global upregulation of pro-inflammatory cytokines, chemokines and angiogenic mediators, and the downregulation of selected regulatory and apoptotic ligands. This pattern reflects a state of chronic immune activation, vascular remodeling and tumor–immune cell crosstalk consistent with advanced metastatic progression. The elevation of multiple inflammatory and angiogenic proteins, alongside the suppression of immune checkpoint–related factors, delineates a circulating immune reprogramming that distinguishes patients with mBC from HDs.

### CTCs correlate with enhanced Treg proportion and pro-metastatic protein enrichment in mBC

To investigate whether CTC presence reflects specific immune alterations, we compared circulating markers, including immune cell populations (Table S3) and circulating proteins (Table S4) in function of the patients’ CTC status (negative vs. positive).

**Fig. 6:**
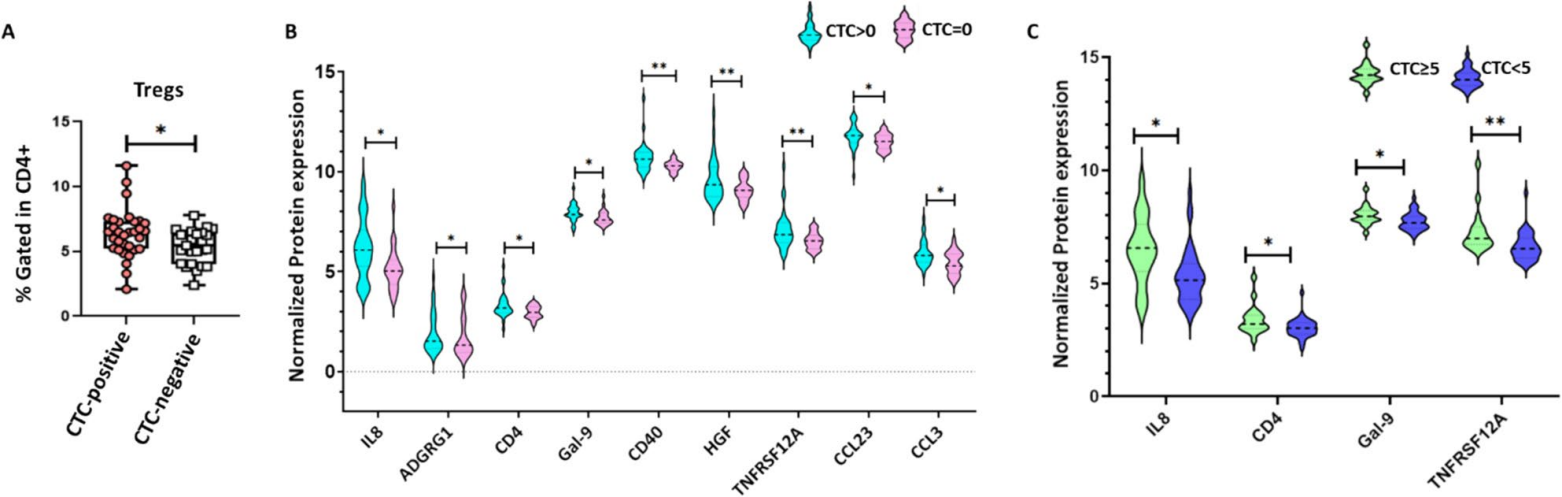
CTCs correlate with enhanced Treg-mediated immune evasion and pro-metastatic protein enrichment in mBC. **A** Comparison of the proportion of Treg cells (CD4⁺CD25⁺CD127^low^) in patients with mBC divided in function of the detection or not of CTCs in their blood sample. **B** Violin plots showing the normalized plasma protein expression levels of IL-8, ADGRG1, CD4, galectin-9, CD40, HGF, TNFRSF12A, CCL23, and CCL3 in CTC-positive and CTC-negative patients. **C** Box and violin plots showing the normalized expression levels of IL-8, galectin-9, CD4, and TNFRSF12A in patients with ≥ 5 CTCs and < 5 CTCs. * *p* < 0.05, **p < 0.01 (nonparametric Wilcoxon–Mann–Whitney test)

This analysis revealed significant immune cell differences. Notably, the proportion of Tregs was significantly higher in the CTC-positive group (*n* = 35 patients) than in the CTC-negative group (*n* = 25 patients) (*p* = 0.034; Fig. 6A), suggesting that CTC presence is associated with enhanced Treg-mediated immunosuppression.

Similarly, several circulating proteins identified in the plasma proteomic analysis were significantly enriched in the CTC-positive group: IL-8, ADGRG1, CD4, galectin-9, CD40, HGF, TNFRSF12A, CCL23, and CCL3 (Fig. 6B). Moreover, in the CTC-positive group IL-8, CD4, galectin-9, and TNFRSF12A were consistently higher in patients with ≥ 5 CTCs than with < 5 CTCs (Fig. 6C). These results point to a dual mechanism in CTC-positive patients, characterized by Treg expansion and selective enrichment of immunomodulatory proteins.

### Association of clinical and biological features with PFS and OS in mBC

**Fig. 7:**
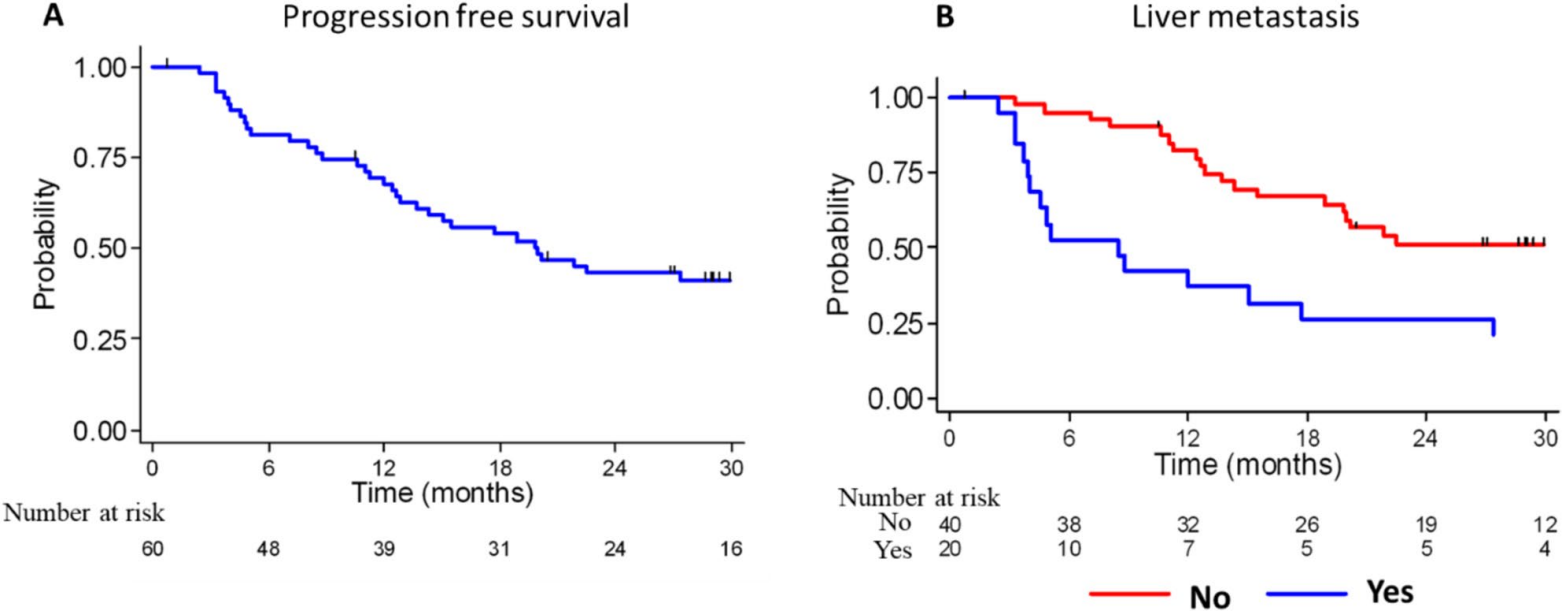
Kaplan–Meier progression-free survival analysis. **A** Kaplan–Meier estimate of progression-free survival in the whole patient cohort (*n* = 60 patients with mBC). **B** Kaplan–Meier survival curves stratified according to the presence or not of liver metastasis. Numbers at risk are shown below each plot

After a median follow-up of 31.5 months (95% CI, 29.9–33.6), the median OS was not reached, and the estimated 30-month OS rate was 20%. The median PFS was 19.9 months (95% CI, 12.8–35.2) (Fig. 7A).

Univariate analyses to evaluate the association between clinical and biological features and PFS did not highlight any significant association with age, histological subtype, tumor grade, or prior systemic breast cancer treatments (neoadjuvant, adjuvant chemotherapy, radiotherapy, or hormone therapy) (Table S5). Conversely, PFS was longer in patients with synchronous metastases than metachronous metastases (HR = 0.39; 95% CI, 0.18–0.84; *p* = 0.010), and shorter in patients with liver metastases (HR = 2.86; 95% CI, 1.45–5.66; *p* = 0.004, Fig. 7B). Bone or lung involvement did not affect PFS.

Among the tumor molecular features, hormone receptor expression (estrogen and/or progesterone receptors) remained a major prognostic factor. Lack of estrogen receptor (HR = 3.01; 95% CI, 1.22–7.42; *p* = 0.032) and progesterone receptor expression (HR = 2.46; 95% CI, 1.25–4.84; *p* = 0.010) was associated with poorer PFS. Conversely, hormone receptor expression was protective (HR = 0.32; 95% CI, 0.12–0.85; *p* = 0.043). HER2 status alone was not correlated with outcome (*p* = 0.891). PFS tended to be poorer in patients with triple-negative tumors (HR⁻/HER2⁻) than with HR⁺/HER2⁻ tumors (HR = 9.66; 95% CI, 1.97–47.42; *p* = 0.087). The presence of at least one detectable CTC was associated with a 2.6-fold higher risk of progression (HR = 2.62; 95% CI, 1.26–5.45; *p* = 0.007), and the presence of ≥ 5 CTCs further increased the risk (HR = 2.20; 95% CI, 1.14–4.25; *p* = 0.021).

Univariate analyses (Table S6) did not highlight any significant association with immune cell subsets. Conversely, elevated plasma levels of IL-8, ADGRG1, NOS3, HGF, TNFRSF12A, ANGPT2, CXCL13, and CSF-1 were associated with shorter PFS (all *p* < 0.05). The strongest associations were observed for IL-8 (HR = 1.62; *p* < 0.001) and NOS3 (HR = 6.66; *p* < 0.001). Conversely, higher circulating levels of TWEAK (HR = 0.33; 95% CI, 0.13–0.82; *p* = 0.022) and FASLG (HR = 0.44; 95% CI, 0.22–0.90; *p* = 0.026) were correlated with longer PFS, although their expression levels were globally lower in patients with mBC than HDs.

In the multivariate Cox regression model (Table 2), IL-8 (HR = 1.49; 95% CI, 1.14–1.95; *p* = 0.004) and NOS3 (HR = 4.72; 95% CI, 1.70–13.15; *p* = 0.003) remained independent predictors of shorter PFS. The presence of liver metastases showed a trend toward an unfavorable outcome (HR = 2.29; *p* = 0.061) as well as triple-negative receptor status (HR = 10.86; *p* = 0.074). Altogether, these results demonstrate that both clinical features (metastatic pattern and tumor subtype) and circulating markers (particularly, IL-8 and NOS3) influence disease progression. The independent prognostic value of IL-8 and NOS3 highlights their potential as biomarkers of aggressive mBC.

**Table 2:** Multivariate Cox proportional hazards analysis of clinical and molecular variables associated with progression-free survival in patients with mBC

Variable	HR^1^	95% CI^1^	*P*-value
Liver metastasis			0.061
No	1	—	
Yes	2.28903	0.9876572, 5.30514	
Breast cancer subtype			0.074
HR+/HER2-	1	—	
HR+/HER2+	1.143714	0.2386535, 5.481092	
HR-/HER2+	2.95561	0.7871169, 11.09826	
HR-/HER2-	10.86004	1.98357, 59.45871	
IL-8	1.490365	1.136743, 1.953994	0.004
NOS3	4.724821	1.69808, 13.14657	0.003

^*1*^*HR* Hazard Ratio, *CI* Exact Confidence Interval

## Discussion

This study provides an integrated cellular and proteomic characterization of the systemic immune alterations in liquid biopsies of patients with mBC. By integrating immune cell profiling, plasma proteomics and CTC enumeration with survival outcomes, we reveal a systemic immune dysfunction linked to metastatic burden, CTC number, and early progression.

Our cohort was predominantly composed of patients with HR⁺/HER2⁻ mBC who received endocrine therapy and CDK4/6 inhibitors, thus mirroring the current therapeutic landscape of advanced luminal breast cancer. Over half of the patients experienced disease progression within two years, underscoring the therapy resistance issue in this molecular subtype. The metastasis localization (mainly bone and liver) aligns with established metastatic tropisms in luminal disease [25, 26]. Moreover, CTC detection in more than half of patients reinforces their prognostic value as a surrogate of metastatic dissemination and systemic disease activity [27–29].

Before initiation of first-line metastatic treatment, patients with mBC exhibited a markedly altered circulating immune landscape compared with HDs. We observed a reduction in total leukocytes (CD45⁺ cells) and within this population, a significant decrease in circulating T cells (CD3⁺) in patients with mBC compared with HDs. This contraction of the T-cell pool without a shift in the CD4⁺ and CD8⁺ populations suggest a broad attrition of effector lymphocyte content in the blood compartment rather than selective lineage skewing. A reduction in circulating T cells in cancer has been linked to chronic antigen stimulation and effector cell compartmentalization in the tumor and metastatic niches, where they may undergo terminal exhaustion [30, 31]. The present data extend this paradigm to untreated mBC, strongly suggesting that systemic immune erosion is not only a consequence of multiple lines of therapy or late-stage cachexia, but an early and intrinsic component of the metastatic state.

Besides quantitative changes, we found qualitative evidence of pan T-cell dysfunction. The proportion of circulating CD3⁺, CD4⁺, and CD8⁺ T cells that expressed inhibitory receptors (PD-1, CTLA-4, LAG-3, and TIGIT) was increased in patients with mBC, compared with HDs. Co-expression of different immune checkpoint molecules in the same T-cell pool is a hallmark of chronic antigen exposure and exhausted, functionally blunted T-cell states, characterized by reduced cytokine production, impaired cytotoxicity, and failure to proliferate upon restimulation [32] .

We also observed a significant Treg expansion in patients with mBC. This suggests an active tolerogenic program rather than a passive collapse of immunity. Importantly, the proportion of Tregs that expressed PD-1, CTLA-4, TIGIT (not significant for LAG-3) was increased in patients with mBC (vs. HDs), indicating that they are phenotypically adapted for potent suppressive function. This coordinated enrichment of immunoregulatory subsets in the blood, in parallel with effector T-cell attrition, supports the view that mBC is sustained by systemic immune reprogramming and not only by local immune escape at metastatic sites [33].

Together, these data challenge the classical view that HR^+^ mBC, which represented the majority in our cohort, is “immunologically cold.” Although these tumors have been considered poorly immunogenic, recent studies reported impressive clinical response to immune checkpoint inhibitors in neoadjuvant settings (alone or in combination with chemotherapy) [34]. Our findings reveal a circulating immune system that is highly engaged, but durably suppressed. This suggests that the limited efficacy of immunotherapy in most mBC subtypes may not reflect a true absence of immune involvement, but rather the dominance of a multi-layered inhibitory control. Particularly, TIGIT emerges as a recurrent inhibitory node in both T cells and NK cells, and consequently as a candidate target for combination immunotherapy in mBC, including in patients who would not traditionally be considered for immunotherapy. This highlights the need to identify strong biomarker(s) to select patients who are likely to respond to immune checkpoint inhibitors.

NK cells further illustrated the paradox of “activated but restrained” immunity in mBC [35]. We found that NK cells (CD56⁺) were significantly increased in patients with mBC compared with HDs. This expansion could be interpreted as a compensatory anti-tumor response. However, we also observed the concurrent enrichment of TIGIT-expressing NK cells in patients with mBC. TIGIT is a major inhibitory receptor in NK cells, and its upregulation is associated with impaired degranulation and reduced cytotoxic function. This suggests that NK cell effector capacity is blunted in mBC. As NK cells play a key role in immune surveillance against CTCs, particularly in the bloodstream and in the pre-metastatic niche [35, 36], the systemic shift toward TIGIT⁺ NK cells provide a plausible mechanism of how CTCs can survive mechanical and immune stress in the vasculature, and ultimately seed distant organs.

The systemic immune alterations were not limited to the lymphoid compartments. Circulating myeloid cells also showed features consistent with tumor-promoting immunoregulation [37]. We observed a significant enrichment in CD163⁺CD206⁺ monocyte-like cells in patients with mBC. These cells are classically described as alternatively activated, or M2-like, monocyte/macrophages with anti-inflammatory, tissue-repair, and pro-tumoral functions. Conversely, the proportion of monocyte/macrophages that expressed CD80 and CD86, markers associated with antigen presentation and T-cell co-stimulation, and often categorized as M1-like cells, tended to be lower (not significant) in patients with mBC. This systemic skewing of the monocyte/macrophage pool toward an immunosuppressive, pro-remodeling phenotype indicates that tumor-promoting myeloid reprogramming is not confined to the metastatic lesion, but is detectable in the circulation. This bias towards circulating M2-like cells and the global T-cell dysfunction and NK cell restraint contribute to an immune ecosystem that supports metastatic outgrowth.

Plasma profiling revealed a coordinated inflammatory, angiogenic, and immunoregulatory signature in mBC. Specifically, the circulating levels of cytokines and chemokines linked to tumor invasion, stromal remodeling and immune suppression, including IL-6, IL-8, CXCL1, CXCL9, CCL3, CCL19, CCL23, CXCL13, and CSF1, were increased in patients with mBC compared with HDs. Several factors associated with vascular remodeling and metastatic colonization, such as HGF, ANGPT2, TNFRSF12A and MMP12, were also increased. Conversely, ligands associated with apoptosis induction and cytotoxic co-stimulation, including FASLG and TWEAK/TNFSF12, were reduced in patients with mBC. Importantly, higher circulating levels of TWEAK and FASLG were correlated with longer PFS. CSF1, which was elevated in patients with mBC, is a driver of survival and polarization of CD163⁺CD206⁺ monocyte/macrophages, linking plasma composition to the observed myeloid remodeling. IL-8, which also was increased, is a chemokine with pleiotropic pro-metastatic roles: it enhances motility, promotes epithelial-to-mesenchymal transition, supports angiogenesis, and recruits myeloid cells that contribute to suppress the anti-tumor immunity. ANGPT2- and NOS3-associated signaling converge on endothelial activation, vascular permeability and niche conditioning in distant organs, especially in the liver and other highly vascularized metastatic targets. The downregulation of FASLG and TWEAK/TNFSF12 suggests systemic attenuation of cytotoxic, death receptor–mediated tumor control. Altogether, the proteomic data describe a host environment that is chronically inflamed, pro-angiogenic and immunosuppressed, a milieu that is permissive to metastatic seeding and expansion.

The coexistence of elevated inflammatory mediators and immunosuppressive myeloid cell polarization is not contradictory, but rather reflects a coordinated tumor-driven systemic reprogramming. In cancer, chronic inflammation frequently promotes the recruitment, survival and alternative activation of monocytes/macrophages through cytokines and growth factors, such as IL-6, IL-8 and CSF1, which are known to drive differentiation toward CD163⁺CD206⁺ immunosuppressive phenotypes [38]. These cells contribute to immune suppression by inhibiting T-cell activation, producing anti-inflammatory mediators and promoting tissue remodeling and angiogenesis. In parallel, inflammatory chemokines and endothelial activation signals facilitate leukocyte trafficking, vascular permeability and metastatic niche formation [8]. Therefore, rather than representing opposing processes, systemic inflammation and immunosuppressive myeloid polarization might cooperate to establish a permissive immunovascular environment that favors CTC survival, endothelial conditioning, and metastatic colonization.

CTCs provide a functional readout of metastatic competence [39, 40]. In our cohort, CTCs were detected in ~ 58% of patients, and their presence and abundance were tightly coupled to features of immune escape. Specifically, the proportion of circulating Tregs was higher in CTC-positive patients. This is consistent with the idea that successful intravasation and survival of tumor cells in blood is not just explained by the tumor cell features but also by the host immune context. These findings suggest that systemic immune imbalance is associated with CTC presence. An immune environment characterized by T-cell exhaustion, Treg expansion, TIGIT-mediated NK dysfunction and M2-like myeloid polarization is likely to favor the survival of CTCs with immune-evasive and stress-resistant phenotypes. Conversely, a more immunocompetent environment may restrict CTC survival or facilitate their selective elimination [41]. Therefore, the heterogeneity in CTC burden and potentially CTC phenotypes may reflect not only the tumor-intrinsic properties but also the degree and nature of systemic immune reprogramming.

The plasma proteomic profile in CTC-positive patients (vs. CTC-negative patients) corroborated this hypothesis, showing enrichment of IL-8, HGF, galectin-9, CD40, TNFRSF12A and the chemokines CCL23 and CCL3, particularly in patients with ≥ 5 CTCs. Many of these factors have roles in promoting chemotaxis, endothelial activation, vascular permeability and resistance to apoptosis. IL-8 has been linked to epithelial-mesenchymal transition, neutrophil recruitment, angiogenesis, and metastatic seeding. HGF/MET signaling supports motility, invasion, and survival in hostile environments, such as the bloodstream. Galectin-9 is implicated in T-cell exhaustion and Treg induction. TNFRSF12A/Fn14 signaling promotes tissue remodeling, fibrosis-like niche conditioning and resistance to therapy. Altogether, these data suggest that the plasma proteomic profile of CTC-positive patients is immunosuppressive, pro-angiogenic and proteolytically active, and fosters dissemination competence. The association of CTC detection and presence of ≥ 5 CTCs with higher risk of early progression (univariate analysis) reinforces the prognostic significance of this axis.

Lastly, and perhaps most importantly from a translational perspective, we identified two soluble factors (IL-8 and NOS3) that independently predict PFS. High baseline IL-8 was associated with shorter PFS, and this association remained significant after adjustment for clinical covariates in the multivariate analysis. IL-8 has been implicated in endocrine resistance, CDK4/6 inhibitor resistance, and metastatic plasticity in HR^+^ breast cancer. Importantly, the majority of patients in this study received endocrine therapy and CDK4/6 inhibitors as first-line treatment [42]. NOS3 regulates nitric oxide production in endothelial cells and thus influences vascular tone, permeability, and angiogenesis. Elevated NOS3 expression was correlated with markedly shorter PFS and remained an independent predictor in the multivariate model. This suggests that IL-8 and NOS3 are not simply surrogate markers of tumor burden or subtype, but capture elements of metastatic biology, specifically inflammatory dissemination and vascular priming, that are not represented by traditional clinicopathologic descriptors.

Clinically, these observations argue for a shift in how we should conceptualize mBC. Instead of viewing it exclusively through the lens of receptor status, lesion distribution and a sequence of systemic therapies, our data support an expanded model in which mBC is a systemic immunovascular disease. In this model, early progression is driven by the intrinsic tumor aggressiveness (e.g., triple-negative phenotype, liver involvement) and also by a permissive host state characterized by exhausted, checkpoint molecule-expressing T cells, numerically expanded but TIGIT-restrained NK cells, monocytes polarized toward the CD163⁺CD206⁺ immunosuppressive phenotype, and a circulating proteomic profile that promotes angiogenesis, tissue remodeling and immune escape. In this model, IL-8 and NOS3 emerge as readily accessible blood-based biomarkers of the permissive immune state and of the risk of rapid progression independently of classical variables. This has immediate implications for patient management. First, IL-8 and NOS3 could be incorporated into the baseline risk assessment to identify patients with a high-risk immunovascular phenotype who might benefit from closer monitoring or intensified upfront combination strategies. Second, these markers, together with TIGIT expression in the T and NK cell compartments and the expansion of suppressive monocyte-like cells, provide a mechanistic rationale for therapeutic strategies that go beyond direct tumor targeting. Such strategies might include simultaneous modulation of inflammatory factor trafficking (e.g. IL-8/CXCR1–CXCR2 blockade), vascular conditioning (targeting NOS3-driven endothelial activation or ANGPT2/HGF signaling), Treg suppression, monocyte repolarization, and multi-checkpoint inhibition, including TIGIT. The present work does not claim that these approaches are ready for immediate clinical application. It demonstrates that their targets are already active and clinically meaningful at the time of metastatic disease detection.

### Limitations and future directions

This study has several limitations. The cohort was modest in size (*n* = 60 total, *n* = 56 for the multivariate analysis), and the follow-up for OS was not reached at the time of analysis. As this study was based on a single prospective cohort, the identification of IL-8 and NOS3 as independent predictors of PFS should be considered hypothesis-generating. Validation in a larger, independent cohort is required to confirm the independent prognostic value of IL-8 and NOS3 and to test their robustness across breast cancer subtypes, treatment types, and metastatic disease sites (visceral vs. bone-predominant vs. lymph nodes). Importantly, biomarker performance may vary according to the molecular subtype, metastatic burden and therapeutic regimen, all of which can influence systemic immune and vascular states. Therefore, external validation in an independent cohort is essential before clinical implementation.

Our analyses are correlative and causality between, for example, TIGIT⁺ NK-cell enrichment and CTC survival, or between NOS3 elevation and liver colonization, cannot be demonstrated. Functional assays (e.g., NK cytotoxicity against autologous CTCs, endothelial permeability assays in patients with plasma with high NOS3 expression) will be essential to bridge mechanism and prognostic observations. Moreover, we focused on peripheral blood. This is a strength in terms of clinical accessibility, but it omits spatial information from the metastatic niche. Future work should integrate paired metastatic biopsies, spatial transcriptomics, or circulating tumor DNA to map how soluble mediators and immune cell subsets in blood correspond to the on-site immunobiology in bone, liver and lung lesions.

Lastly, treatment heterogeneity (most patients received endocrine therapy plus CDK4/6 inhibition, but others received chemotherapy ± targeted therapy or immunotherapy) could modulate disease progression dynamics. It is known that the different therapeutic regimens can exert distinct and sometimes divergent effects on circulating immune cell subsets, cytokine profiles, endothelial activation and CTC dynamics. Indeed, endocrine therapy and CDK4/6 inhibitors may partially restore immune activation in luminal disease [43]. Conversely, chemotherapy can induce immunogenic cell death and systemic lymphocyte depletion, and immune checkpoint inhibitors may selectively reverse exhaustion programs [44]. Due to the limited sample size of each treatment subgroup, stratified survival analyses according to treatment type would have been underpowered and therefore, were not performed. Larger cohorts with balanced treatment subgroups are needed to formally assess whether IL-8, NOS3, CTC burden, or immune exhaustion markers retain prognostic value independently of the therapeutic regimens. Longitudinal sampling to capture how IL-8, NOS3, TIGIT⁺ NK cells and Treg burden change under specific regimens will be crucial to determine whether these markers are static prognostic indicators or dynamic pharmacodynamic readouts.

## Conclusions

**Fig. 8:**
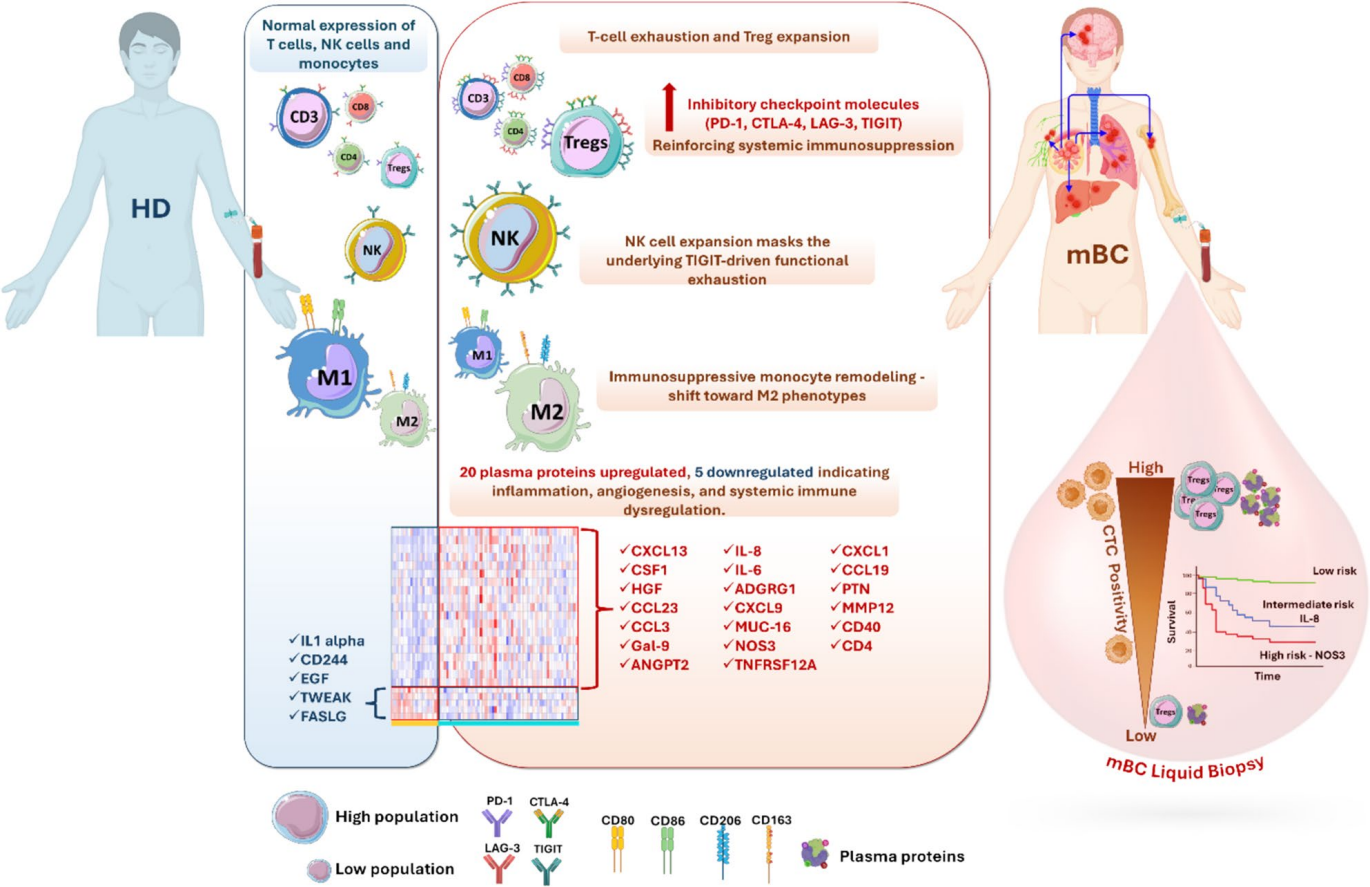
Systemic immune and proteomic remodeling in patients with mBC. Summary illustration showing the main circulating immune alterations in mBC. Compared with HDs, patients with mBC exhibit T-cell exhaustion and Treg expansion, marked by high expression of inhibitory immune checkpoints (PD-1, CTLA-4, LAG-3, TIGIT). NK cells are numerically increased, but many express TIGIT, indicating functional inhibition. Circulating monocytes show a shift toward the immunosuppressive M2-like phenotype (CD163⁺CD206⁺), reflecting myeloid remodeling. Plasma proteomic profiling revealed a distinct circulating signature in mBC composed of 20 upregulated (including IL-6, IL-8, CXCL9, ANGPT2, CSF1) and 5 downregulated proteins (IL-1α, CD244, EGF, TWEAK, FASLG), indicating systemic inflammation, angiogenesis and immune dysregulation. These data highlight a coordinated remodeling of circulating immune cells and proteins in mBC, supporting a global shift toward immune suppression and a tumor-promoting systemic environment

This work defines mBC at first detection as a state of systemic immune dysregulation characterized by (i) depletion of global T-cell content and expansion of highly suppressive, checkpoint factor-expressing Tregs; (ii) effector T and NK cells that remain present in circulation, but are deeply inhibited through multi-checkpoint receptor expression, notably TIGIT; (iii) circulating monocytes/macrophages skewed toward immunoregulatory, tumor-supportive phenotypes; and (iv) a soluble inflammatory/angiogenic proteomic profile marked by elevated concentration of IL-8, NOS3 and other mediators of vascular remodeling, invasion and immune suppression (Fig. 8).

These features are not merely descriptive. They converge tightly with CTC burden and independently predict early progression. IL-8 and NOS3 emerge as clinically accessible prognostic markers that outperform several traditional clinicopathologic factors in multivariate analysis. Together, our data argue that mBC should be viewed and eventually managed as a systemic immunovascular disease in which tumor cell dissemination, immune escape, and organ colonization are molecularly interconnected. This opens the door to blood-based risk stratification and to therapeutic strategies that go beyond tumor cell–intrinsic targeting to actively reprogram the host environment that allows metastatic disease to persist.

## Supplementary Information

Supplementary Material 1.

## Data Availability

The datasets generated and analyzed during the current study are included in this published article and its supplementary information files.
