# Supplementary material for: Liquid biopsy reveals the immune status and protein profiles linked to CTC burden and clinical outcomes in metastatic breast cancer
Source: J Exp Clin Cancer Res. 2026 Apr 18;45:111. doi: 10.1186/s13046-026-03709-3 (PMC13154879; doi:10.1186/s13046-026-03709-3)
Supplement: Supplementary file 1 — Supplementary Material 1. [file 13046_2026_3709_MOESM1_ESM.docx]

**SUPPLEMENTARY MATERIALS**

**Liquid biopsy reveals the immune status and protein profiles linked to CTC burden and clinical outcomes in metastatic breast cancer**

**Fig S1. Overview of the mBC patient cohort and experimental workflow.**


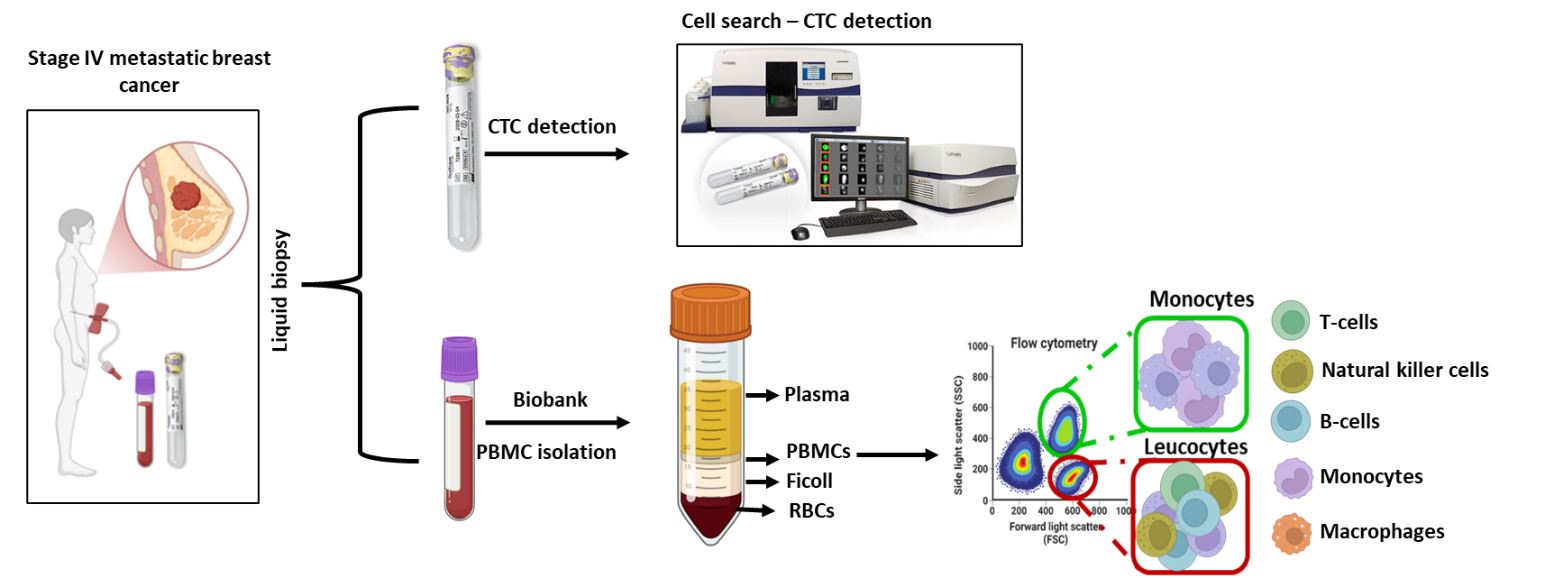


**Fig S1. Overview of the mBC patient cohort and experimental workflow.** Peripheral blood samples were collected from patients with stage IV mBC before first-line treatment initiation. Blood was processed to isolate CTCs and PBMCs. Downstream analyses included CTC enumeration using the FDA-approved CellSearch^®^ system and PBMC immune phenotyping by flow cytometry. RBC, Red blood cells.

**Table S1: Patients’ characteristics**

| Variables |  | Total n, (%) |
| --- | --- | --- |
| Patients |  | 60 |
| Age (years), median (range) | | 63.5 (33-89) |
| Metastatic disease features | |  |
|  | Synchronous metastases | 23 (38) |
|  | Metachronous metastases | 37 (62) |
|  |  |  |
|  | Time from initial breast cancer diagnosis to inclusion (months), median (range) | 40 (0-366) |
|  |  |  |
|  | Number of metastatic sites, mean(range) | 2 (1-6) |
|  | Multiple metastatic sites | 34 (56.7) |
|  | Metastatic sites |  |
|  | Bone | 37 (62) |
|  | Liver | 20 (33) |
|  | Lung | 17 (28) |
|  | Lymph nodes | 15 (25) |
| Histological type | |  |
|  | Ductal invasive carcinoma | 50 (83) |
|  | Lobular carcinoma | 10 (17) |
| Histological (SBR) grade | |  |
|  | 1 | 7 (14) |
|  | 2 or 3 | 44 (86) |
|  | Missing | 9 |
| Molecular subtypes | |  |
|  | HR+/HER2**-** | 50 (84) |
|  | HR+/HER2+ | 5 (8) |
|  | HR-/HER2+ | 3 (5) |
|  | HR**-**/HER2**-** | 2 (3) |
| CTC analysis at diagnosis | |  |
|  | CTC enumeration, mean (range) | 31 (0-962) |
|  | ≥1 CTC/7.5mL | 35 (58) |
|  | ≥5 CTC/7.5mL | 21 (35) |
|  | ≥1 CTC-AXL/7.5mL | 7 (12) |

**Table S2: Comparison of circulating immune cell populations in HDs (n=21) and patients with mBC (n=60).**

| **Variable** | **HDs  N = 21** | **mBC  N = 60** | **p-value** | **q-value^1^** |
| --- | --- | --- | --- | --- |
| **% Gated CD45^+^** |  |  | **0.001^2^** | **0.007** |
| Mean (± SD) | 88.20 (± 7.15) | 77.44 (± 14.73) |  |  |
| Median (Q1; Q3) | 90.99 (84.74; 93.32) | 78.65 (69.15; 87.46) |  |  |
| [Min; Max] | [72.72; 96.61] | [18.03; 97.96] |  |  |
| Missing | 0 | 5 |  |  |
| **% Gated CD45^+^/CD3^+^** |  |  | **<0.001^2^** | **<0.001** |
| Mean (± SD) | 79.52 (± 5.37) | 67.25 (± 11.15) |  |  |
| Median (Q1; Q3) | 80.89 (76.23; 82.01) | 69.91 (63.86; 74.65) |  |  |
| [Min; Max] | [66.77; 89.97] | [23.66; 84.57] |  |  |
| Missing | 0 | 5 |  |  |
| **% Gated CD45^+^/CD3^-^** |  |  | **<0.001^2^** | **<0.001** |
| Mean (± SD) | 20.13 (± 5.53) | 32.22 (± 10.97) |  |  |
| Median (Q1; Q3) | 18.68 (16.69; 23.80) | 29.76 (24.98; 35.33) |  |  |
| [Min ; Max] | [9.93 ; 33.37] | [14.84 ; 76.09] |  |  |
| Missing | 0 | 5 |  |  |
| **% Gated CD3^+^/CD4^+^** |  |  | 0.250^2^ | 0.406 |
| Mean (± SD) | 69.81 (± 8.37) | 67.13 (± 9.90) |  |  |
| Median (Q1 ; Q3) | 72.67 (62.35 ; 76.97) | 68.89 (58.28 ; 74.59) |  |  |
| [Min ; Max] | [55.32 ; 81.89] | [49.10 ; 88.75] |  |  |
| Missing | 0 | 5 |  |  |
| **% Gated CD3^+^/CD8^+^** |  |  | 0.326^2^ | 0.483 |
| Mean (± SD) | 22.34 (± 7.14) | 24.03 (± 7.90) |  |  |
| Median (Q1 ; Q3) | 20.60 (16.14 ; 27.96) | 23.29 (18.03 ; 29.02) |  |  |
| [Min ; Max] | [12.82 ; 34.47] | [7.13 ; 41.59] |  |  |
| Missing | 0 | 5 |  |  |
| **% Gated CD3^+^/CD4^+^CD8^+^** |  |  | 0.949^2^ | 0.981 |
| Mean (± SD) | 4.14 (± 1.83) | 5.10 (± 4.22) |  |  |
| Median (Q1 ; Q3) | 3.72 (2.97 ; 5.74) | 3.74 (2.38 ; 6.90) |  |  |
| [Min ; Max] | [0.79 ; 7.16] | [0.78 ; 22.92] |  |  |
| Missing | 0 | 5 |  |  |
| **% Gated CD3^+^/CD4^-^CD8^-^** |  |  | 0.977^2^ | 0.983 |
| Mean (± SD) | 3.38 (± 1.76) | 3.73 (± 2.54) |  |  |
| Median (Q1 ; Q3) | 2.95 (2.50 ; 3.83) | 2.99 (1.93 ; 4.77) |  |  |
| [Min ; Max] | [1.18 ; 9.46] | [0.81 ; 12.48] |  |  |
| Missing | 0 | 5 |  |  |
| **% Gated CD3^+^/PD1^+^** |  |  | **<0.001^2^** | **<0.001** |
| Mean (± SD) | 1.60 (± 1.49) | 5.48 (± 4.26) |  |  |
| Median (Q1 ; Q3) | 1.23 (0.19 ; 2.46) | 4.74 (1.54 ; 8.64) |  |  |
| [Min ; Max] | [0.07 ; 4.76] | [0.21 ; 15.26] |  |  |
| Missing | 0 | 5 |  |  |
| **% Gated CD3^+^ / CTLA4^+^** |  |  | **0.002^2^** | **0.010** |
| Mean (± SD) | 3.95 (± 5.50) | 17.29 (± 19.91) |  |  |
| Median (Q1 ; Q3) | 0.72 (0.29 ; 7.12) | 9.57 (0.82 ; 22.11) |  |  |
| [Min ; Max] | [0.22 ; 19.46] | [0.21 ; 72.98] |  |  |
| Missing | 0 | 5 |  |  |
| **% Gated CD3^+^/LAG3^+^** |  |  | **0.013^2^** | **0.042** |
| Mean (± SD) | 61.14 (± 11.85) | 69.46 (± 13.56) |  |  |
| Median (Q1 ; Q3) | 63.81 (49.77 ; 68.43) | 70.63 (61.47 ; 79.94) |  |  |
| [Min ; Max] | [40.83 ; 87.34] | [36.71 ; 92.43] |  |  |
| Missing | 0 | 5 |  |  |
| **% Gated CD3^+^/TIGIT^+^** |  |  | **<0.001^2^** | **0.002** |
| Mean (± SD) | 31.92 (± 19.23) | 58.69 (± 29.16) |  |  |
| Median (Q1 ; Q3) | 35.70 (12.73 ; 49.43) | 63.37 (27.60 ; 85.28) |  |  |
| [Min ; Max] | [6.80 ; 59.60] | [9.98 ; 95.40] |  |  |
| Missing | 0 | 5 |  |  |
| **% Gated CD4^+^/TREG^+^** |  |  | **<0.001^2^** | **<0.001** |
| Mean (± SD) | 3.38 (± 0.73) | 5.97 (± 1.71) |  |  |
| Median (Q1 ; Q3) | 3.24 (3.01 ; 3.93) | 6.07 (5.11 ; 6.76) |  |  |
| [Min ; Max] | [1.88 ; 4.71] | [2.09 ; 11.61] |  |  |
| Missing | 0 | 5 |  |  |
| **% Gated CD4^+^/PD1^+^** |  |  | **<0.001^2^** | **<0.001** |
| Mean (± SD) | 1.52 (± 1.38) | 5.46 (± 4.44) |  |  |
| Median (Q1 ; Q3) | 1.36 (0.23 ; 2.12) | 4.36 (1.66 ; 8.20) |  |  |
| [Min ; Max] | [0.05 ; 4.21] | [0.23 ; 18.22] |  |  |
| Missing | 0 | 5 |  |  |
| **% Gated CD4^+^/CTLA4^+^** |  |  | **0.002^2^** | **0.009** |
| Mean (± SD) | 2.95 (± 4.42) | 14.93 (± 19.39) |  |  |
| Median (Q1 ; Q3) | 0.65 (0.20 ; 4.08) | 7.57 (0.72 ; 17.60) |  |  |
| [Min ; Max] | [0.16 ; 17.65] | [0.17 ; 76.58] |  |  |
| Missing | 0 | 5 |  |  |
| **% Gated CD4^+^/LAG3^+^** |  |  | **0.015^2^** | **0.044** |
| Mean (± SD) | 56.98 (± 13.01) | 65.90 (± 14.53) |  |  |
| Median (Q1 ; Q3) | 60.43 (42.50 ; 65.67) | 67.14 (55.42 ; 76.57) |  |  |
| [Min ; Max] | [35.45 ; 86.39] | [30.86 ; 91.91] |  |  |
| Missing | 0 | 5 |  |  |
| **% Gated CD4^+^/TIGIT^+^** |  |  | **<0.001^2^** | **0.002** |
| Mean (± SD) | 24.73 (± 18.56) | 52.83 (± 31.26) |  |  |
| Median (Q1 ; Q3) | 25.38 (5.88 ; 45.21) | 57.69 (20.53 ; 83.10) |  |  |
| [Min ; Max] | [3.41 ; 52.79] | [5.59 ; 93.80] |  |  |
| Missing | 0 | 5 |  |  |
| **% Gated TREG^+^/PD1^+^** |  |  | **<0.001^2^** | **0.001** |
| Mean (± SD) | 1.50 (± 1.70) | 4.99 (± 4.35) |  |  |
| Median (Q1 ; Q3) | 0.94 (0.23 ; 1.72) | 3.25 (1.43 ; 8.06) |  |  |
| [Min ; Max] | [0.09 ; 6.15] | [0.38 ; 15.78] |  |  |
| Missing | 0 | 5 |  |  |
| **% Gated TREG^+^/CTLA4^+^** |  |  | **0.003^2^** | **0.014** |
| Mean (± SD) | 6.46 (± 9.03) | 18.73 (± 21.05) |  |  |
| Median (Q1 ; Q3) | 2.21 (1.77 ; 6.00) | 9.53 (3.24 ; 26.97) |  |  |
| [Min ; Max] | [0.58 ; 31.42] | [0.65 ; 82.67] |  |  |
| Missing | 0 | 5 |  |  |
| **% Gated TREG^+^/LAG3^+^** |  |  | 0.058^2^ | 0.134 |
| Mean (± SD) | 55.01 (± 12.77) | 62.33 (± 14.29) |  |  |
| Median (Q1 ; Q3) | 56.88 (46.70 ; 61.93) | 61.20 (52.01 ; 73.75) |  |  |
| [Min ; Max] | [29.28 ; 79.79] | [28.04 ; 88.67] |  |  |
| Missing | 0 | 5 |  |  |
| **% Gated TREG^+^/TIGIT^+^ (Gated)** |  |  | **<0.001^2^** | **0.003** |
| Mean (± SD) | 43.57 (± 21.89) | 66.93 (± 26.18) |  |  |
| Median (Q1 ; Q3) | 44.95 (21.20 ; 68.17) | 72.84 (41.60 ; 90.96) |  |  |
| [Min ; Max] | [16.21 ; 72.93] | [15.80 ; 97.85] |  |  |
| Missing | 0 | 5 |  |  |
| **% Gated CD8^+^/PD1^+^** |  |  | **<0.001^2^** | **0.001** |
| Mean (± SD) | 1.64 (± 1.86) | 5.24 (± 3.93) |  |  |
| Median (Q1 ; Q3) | 0.99 (0.16 ; 2.66) | 5.25 (1.67 ; 7.89) |  |  |
| [Min ; Max] | [0.07 ; 7.65] | [0.05 ; 13.47] |  |  |
| Missing | 0 | 5 |  |  |
| **% Gated CD8^+^/CTLA4^+^** |  |  | **0.017^2^** | 0.050 |
| Mean (± SD) | 5.63 (± 8.39) | 17.17 (± 18.71) |  |  |
| Median (Q1 ; Q3) | 0.66 (0.20 ; 9.81) | 10.29 (0.60 ; 24.40) |  |  |
| [Min ; Max] | [0.13 ; 27.66] | [0.07 ; 70.87] |  |  |
| Missing | 0 | 5 |  |  |
| **% Gated CD8^+^/LAG3^+^** |  |  | **0.004^2^** | **0.020** |
| Mean (± SD) | 67.23 (± 12.85) | 76.53 (± 11.63) |  |  |
| Median (Q1 ; Q3) | 71.12 (58.11 ; 74.92) | 77.24 (68.81 ; 86.31) |  |  |
| [Min ; Max] | [32.29 ; 90.35] | [46.31 ; 93.65] |  |  |
| Missing | 0 | 5 |  |  |
| **% Gated CD8^+^/TIGIT^+^** |  |  | **<0.001^2^** | **0.001** |
| Mean (± SD) | 47.25 (± 20.86) | 71.77 (± 24.36) |  |  |
| Median (Q1 ; Q3) | 52.04 (26.53 ; 67.46) | 79.12 (52.84 ; 92.28) |  |  |
| [Min ; Max] | [16.90 ; 74.43] | [18.89 ; 98.36] |  |  |
| Missing | 0 | 5 |  |  |
| **% Gated CD8+/DNAM1+** |  |  | **<0.0012** | **<0.001** |
| Mean (± SD) | 79.21 (± 12.49) | 90.79 (± 8.75) |  |  |
| Median (Q1 ; Q3) | 81.01 (69.48 ; 90.04) | 94.06 (87.41 ; 97.08) |  |  |
| [Min ; Max] | [55.24 ; 93.52] | [66.84 ; 99.42] |  |  |
| Missing | 0 | 5 |  |  |
| **% Gated CD3-/CD19+** |  |  | **0.018^2^** | 0.051 |
| Mean (± SD) | 57.11 (± 10.70) | 48.50 (± 14.75) |  |  |
| Median (Q1 ; Q3) | 56.32 (50.71 ; 64.63) | 49.30 (38.77 ; 59.01) |  |  |
| [Min ; Max] | [36.87 ; 75.70] | [3.34 ; 76.08] |  |  |
| Missing | 0 | 5 |  |  |
| **% Gated CD3- / CD56+** |  |  | **<0.001^2^** | **0.003** |
| Mean (± SD) | 21.07 (± 10.75) | 33.37 (± 15.94) |  |  |
| Median (Q1 ; Q3) | 18.37 (14.28 ; 25.69) | 27.81 (22.35 ; 43.32) |  |  |
| [Min ; Max] | [7.67 ; 48.04] | [6.84 ; 77.90] |  |  |
| Missing | 0 | 5 |  |  |
| **% Gated CD56+ / TIGIT+** |  |  | **<0.001^2^** | **0.005** |
| Mean (± SD) | 60.97 (± 24.65) | 77.92 (± 23.17) |  |  |
| Median (Q1 ; Q3) | 74.47 (35.02 ; 83.59) | 89.83 (56.72 ; 94.92) |  |  |
| [Min ; Max] | [18.80 ; 92.53] | [27.77 ; 99.12] |  |  |
| Missing | 0 | 5 |  |  |
| **% Gated CD45+** |  |  | 0.186^2^ | 0.326 |
| Mean (± SD) | 97.60 (± 1.41) | 98.04 (± 1.28) |  |  |
| Median (Q1 ; Q3) | 97.87 (97.28 ; 98.70) | 98.42 (97.36 ; 98.97) |  |  |
| [Min ; Max] | [94.10 ; 99.47] | [92.30 ; 99.34] |  |  |
| Missing | 0 | 4 |  |  |
| **% Gated CD45+ / CD3+** |  |  | 0.427^2^ | 0.592 |
| Mean (± SD) | 29.37 (± 8.96) | 27.28 (± 12.83) |  |  |
| Median (Q1 ; Q3) | 28.14 (23.38 ; 33.36) | 26.88 (18.67 ; 33.61) |  |  |
| [Min ; Max] | [14.83 ; 49.29] | [0.49 ; 56.13] |  |  |
| Missing | 0 | 4 |  |  |
| **% Gated CD45+ / CD3-** |  |  | 0.375^2^ | 0.535 |
| Mean (± SD) | 69.82 (± 9.20) | 72.16 (± 13.03) |  |  |
| Median (Q1 ; Q3) | 71.02 (66.14 ; 75.69) | 72.31 (65.67 ; 80.95) |  |  |
| [Min ; Max] | [49.36 ; 84.75] | [42.87 ; 99.42] |  |  |
| Missing | 0 | 4 |  |  |
| **% Gated CD3- / CD19+** |  |  | 0.317^2^ | 0.481 |
| Mean (± SD) | 15.50 (± 6.69) | 14.22 (± 8.28) |  |  |
| Median (Q1 ; Q3) | 12.93 (11.01 ; 20.79) | 12.57 (8.65 ; 18.01) |  |  |
| [Min ; Max] | [5.55 ; 29.02] | [0.16 ; 39.05] |  |  |
| Missing | 0 | 4 |  |  |
| **% Gated CD3- / CD19-** |  |  | 0.913^2^ | 0.977 |
| Mean (± SD) | 79.01 (± 7.76) | 77.94 (± 11.83) |  |  |
| Median (Q1 ; Q3) | 79.03 (75.79 ; 84.25) | 81.66 (71.97 ; 85.77) |  |  |
| [Min ; Max] | [60.46 ; 91.67] | [39.46 ; 99.50] |  |  |
| Missing | 0 | 4 |  |  |
| **% Gated CD3-CD19- / CD11B-CD14-** |  |  | 0.348^2^ | 0.506 |
| Mean (± SD) | 1.75 (± 2.14) | 2.00 (± 3.05) |  |  |
| Median (Q1 ; Q3) | 1.26 (0.11 ; 2.98) | 1.23 (0.31 ; 2.40) |  |  |
| [Min ; Max] | [0.00 ; 8.59] | [0.00 ; 20.50] |  |  |
| Missing | 0 | 4 |  |  |
| **% Gated CD3-CD19- / CD11B+ / CD14-** |  |  | 0.615^2^ | 0.759 |
| Mean (± SD) | 3.52 (± 5.75) | 2.07 (± 2.59) |  |  |
| Median (Q1 ; Q3) | 0.74 (0.10 ; 3.28) | 1.07 (0.33 ; 2.73) |  |  |
| [Min ; Max] | [0.01 ; 18.86] | [0.00 ; 11.07] |  |  |
| Missing | 0 | 4 |  |  |
| **% Gated CD3-CD19- / CD11B+ / CD14+** |  |  | 0.941^2^ | 0.981 |
| Mean (± SD) | 90.58 (± 7.53) | 91.34 (± 6.87) |  |  |
| Median (Q1 ; Q3) | 91.63 (84.52 ; 97.88) | 92.38 (87.99 ; 97.13) |  |  |
| [Min ; Max] | [76.26 ; 99.44] | [65.72 ; 98.87] |  |  |
| Missing | 0 | 4 |  |  |
| **% Gated CD3-CD19- / CD11B- / CD14+** |  |  | 0.372^2^ | 0.535 |
| Mean (± SD) | 4.15 (± 4.30) | 4.58 (± 4.22) |  |  |
| Median (Q1 ; Q3) | 2.28 (0.76 ; 6.35) | 3.31 (1.27 ; 6.63) |  |  |
| [Min ; Max] | [0.43 ; 15.86] | [0.19 ; 20.72] |  |  |
| Missing | 0 | 4 |  |  |
| **% Gated CD11B+CD14+ / CD80+CD86+** |  |  | 0.172^2^ | 0.307 |
| Mean (± SD) | 29.21 (± 22.30) | 19.60 (± 14.11) |  |  |
| Median (Q1 ; Q3) | 18.67 (9.39 ; 54.77) | 17.07 (7.65 ; 28.68) |  |  |
| [Min ; Max] | [4.05 ; 67.71] | [1.65 ; 58.76] |  |  |
| Missing | 0 | 4 |  |  |
| **% Gated CD11B+CD14+ / CD163+CD206+** |  |  | **<0.001^2^** | **0.004** |
| Mean (± SD) | 27.11 (± 17.39) | 49.91 (± 23.34) |  |  |
| Median (Q1 ; Q3) | 26.99 (12.22 ; 40.80) | 47.49 (29.12 ; 71.72) |  |  |
| [Min ; Max] | [1.75 ; 55.62] | [10.96 ; 89.77] |  |  |
| Missing | 0 | 4 |  |  |
| ^1^False discovery rate correction for multiple testing | | | | |
| ^2^Wilcoxon rank sum test | | | | |

**Table S3: Comparison of circulating immune cell populations in CTC-positive and CTC-negative patients with mBC (N=60: CTC>0 vs CTC=0)**

| **Variable** | **CTC>0  N = 35** | **CTC=0  N = 25** | **p-value** | **q-value^1^** |
| --- | --- | --- | --- | --- |
| **CD45+ (Gated)** |  |  | 0.629^2^ | 0.993 |
| Mean (± SD) | 77.52 (± 16.64) | 77.32 (± 11.93) |  |  |
| Median (Q1 ; Q3) | 80.85 (69.40 ; 88.16) | 77.94 (69.75 ; 86.40) |  |  |
| [Min ; Max] | [18.03 ; 97.96] | [50.24 ; 97.04] |  |  |
| Missing | 3 | 2 |  |  |
| **CD45+ / CD3+ (Gated)** |  |  | 0.366^3^ | 0.705 |
| Mean (± SD) | 67.48 (± 13.03) | 66.93 (± 8.12) |  |  |
| Median (Q1 ; Q3) | 70.29 (64.29 ; 74.83) | 67.59 (63.79 ; 71.02) |  |  |
| [Min ; Max] | [23.66 ; 84.57] | [48.92 ; 78.72] |  |  |
| Missing | 3 | 2 |  |  |
| **CD45+ / CD3- (Gated)** |  |  | 0.267^3^ | 0.556 |
| Mean (± SD) | 31.83 (± 12.71) | 32.77 (± 8.20) |  |  |
| Median (Q1 ; Q3) | 28.64 (24.56 ; 34.65) | 31.94 (28.66 ; 36.10) |  |  |
| [Min ; Max] | [14.84 ; 76.09] | [20.15 ; 51.19] |  |  |
| Missing | 3 | 2 |  |  |
| **CD3+ / PD-1+ (Gated)** |  |  | 0.872^2^ | 0.993 |
| Mean (± SD) | 5.56 (± 4.27) | 5.38 (± 4.35) |  |  |
| Median (Q1 ; Q3) | 4.87 (1.54 ; 8.37) | 4.74 (1.51 ; 9.05) |  |  |
| [Min ; Max] | [0.25 ; 15.26] | [0.21 ; 13.30] |  |  |
| Missing | 3 | 2 |  |  |
| **CD3+ / CTLA-4+ (Gated)** |  |  | 0.688^3^ | 0.993 |
| Mean (± SD) | 17.78 (± 20.54) | 16.61 (± 19.42) |  |  |
| Median (Q1 ; Q3) | 10.71 (3.27 ; 23.28) | 9.23 (0.50 ; 19.81) |  |  |
| [Min ; Max] | [0.21 ; 72.98] | [0.21 ; 57.59] |  |  |
| Missing | 3 | 2 |  |  |
| **CD3+ / LAG-3+ (Gated)** |  |  | 0.176^2^ | 0.459 |
| Mean (± SD) | 67.41 (± 12.96) | 72.31 (± 14.14) |  |  |
| Median (Q1 ; Q3) | 68.48 (61.86 ; 77.06) | 74.15 (61.47 ; 83.36) |  |  |
| [Min ; Max] | [37.09 ; 86.01] | [36.71 ; 92.43] |  |  |
| Missing | 3 | 2 |  |  |
| **CD3+ / TIGIT+ (Gated)** |  |  | 0.980^2^ | 0.993 |
| Mean (± SD) | 59.24 (± 28.47) | 57.91 (± 30.73) |  |  |
| Median (Q1 ; Q3) | 62.82 (34.93 ; 83.47) | 66.99 (26.58 ; 86.27) |  |  |
| [Min ; Max] | [10.91 ; 95.25] | [9.98 ; 95.40] |  |  |
| Missing | 3 | 2 |  |  |
| **CD3+ / DNAM1+ (Gated)** |  |  | 0.859^2^ | 0.993 |
| Mean (± SD) | 90.33 (± 8.66) | 90.01 (± 9.13) |  |  |
| Median (Q1 ; Q3) | 93.74 (89.48 ; 95.89) | 93.80 (85.11 ; 96.48) |  |  |
| [Min ; Max] | [66.71 ; 98.29] | [69.35 ; 99.32] |  |  |
| Missing | 3 | 2 |  |  |
| **CD4+ / Treg+ (Gated)** |  |  | **0.034^3^** | 0.212 |
| Mean (± SD) | 6.37 (± 1.86) | 5.41 (± 1.32) |  |  |
| Median (Q1 ; Q3) | 6.49 (5.25 ; 7.29) | 5.79 (4.52 ; 6.40) |  |  |
| [Min ; Max] | [2.09 ; 11.61] | [2.41 ; 7.77] |  |  |
| Missing | 3 | 2 |  |  |
| **CD4+ / PD-1+ (Gated)** |  |  | 0.720^3^ | 0.993 |
| Mean (± SD) | 5.72 (± 4.68) | 5.09 (± 4.17) |  |  |
| Median (Q1 ; Q3) | 4.80 (1.99 ; 8.07) | 4.25 (1.33 ; 8.62) |  |  |
| [Min ; Max] | [0.24 ; 18.22] | [0.23 ; 13.45] |  |  |
| Missing | 3 | 2 |  |  |
| **CD4+ / CTLA-4+ (Gated)** |  |  | 0.925^3^ | 0.993 |
| Mean (± SD) | 14.74 (± 19.79) | 15.18 (± 19.25) |  |  |
| Median (Q1 ; Q3) | 7.24 (1.67 ; 18.54) | 8.09 (0.38 ; 16.74) |  |  |
| [Min ; Max] | [0.17 ; 76.58] | [0.19 ; 57.73] |  |  |
| Missing | 3 | 2 |  |  |
| **CD4+ / LAG-3+ (Gated)** |  |  | 0.127^2^ | 0.414 |
| Mean (± SD) | 63.54 (± 13.66) | 69.19 (± 15.35) |  |  |
| Median (Q1 ; Q3) | 65.40 (54.35 ; 73.16) | 71.10 (56.09 ; 81.67) |  |  |
| [Min ; Max] | [30.86 ; 84.51] | [31.51 ; 91.91] |  |  |
| Missing | 3 | 2 |  |  |
| **CD4+ / TIGIT+ (Gated)** |  |  | 0.980^2^ | 0.993 |
| Mean (± SD) | 53.19 (± 30.61) | 52.32 (± 32.82) |  |  |
| Median (Q1 ; Q3) | 59.14 (23.10 ; 77.61) | 55.05 (20.31 ; 84.80) |  |  |
| [Min ; Max] | [5.59 ; 93.73] | [5.84 ; 93.80] |  |  |
| Missing | 3 | 2 |  |  |
| **CD4+ / DNAM1+ (Gated)** |  |  | 0.819^2^ | 0.993 |
| Mean (± SD) | 89.66 (± 9.64) | 89.59 (± 9.92) |  |  |
| Median (Q1 ; Q3) | 92.91 (89.03 ; 96.27) | 93.63 (85.16 ; 97.11) |  |  |
| [Min ; Max] | [64.26 ; 98.27] | [67.16 ; 99.28] |  |  |
| Missing | 3 | 2 |  |  |
| **Treg+ / PD-1+ (Gated)** |  |  | 0.701^3^ | 0.993 |
| Mean (± SD) | 5.05 (± 4.04) | 4.90 (± 4.84) |  |  |
| Median (Q1 ; Q3) | 3.77 (1.71 ; 7.90) | 3.25 (0.87 ; 8.18) |  |  |
| [Min ; Max] | [0.38 ; 15.26] | [0.39 ; 15.78] |  |  |
| Missing | 3 | 2 |  |  |
| **Treg+ / CTLA-4+ (Gated)** |  |  | 0.627^3^ | 0.993 |
| Mean (± SD) | 19.33 (± 21.49) | 17.90 (± 20.88) |  |  |
| Median (Q1 ; Q3) | 9.83 (3.86 ; 26.01) | 9.51 (2.70 ; 26.97) |  |  |
| [Min ; Max] | [0.78 ; 82.67] | [0.65 ; 67.14] |  |  |
| Missing | 3 | 2 |  |  |
| **Treg+ / TIGIT+ (Gated)** |  |  | 0.754^2^ | 0.993 |
| Mean (± SD) | 66.71 (± 25.83) | 67.24 (± 27.23) |  |  |
| Median (Q1 ; Q3) | 74.52 (42.73 ; 88.61) | 72.23 (41.60 ; 92.66) |  |  |
| [Min ; Max] | [15.80 ; 97.63] | [24.71 ; 97.85] |  |  |
| Missing | 3 | 2 |  |  |
| **Treg+ / DNAM1+ (Gated)** |  |  | 0.986^3^ | 0.993 |
| Mean (± SD) | 79.87 (± 11.14) | 78.85 (± 14.47) |  |  |
| Median (Q1 ; Q3) | 83.71 (72.07 ; 88.59) | 83.54 (63.59 ; 90.43) |  |  |
| [Min ; Max] | [54.17 ; 93.65] | [53.28 ; 97.34] |  |  |
| Missing | 3 | 2 |  |  |
| **Treg+ / NKG2A+ (Gated)** |  |  | 0.259^2^ | 0.556 |
| Mean (± SD) | 65.70 (± 17.10) | 70.78 (± 14.61) |  |  |
| Median (Q1 ; Q3) | 66.68 (52.02 ; 80.08) | 71.60 (63.09 ; 82.58) |  |  |
| [Min ; Max] | [34.95 ; 91.71] | [33.46 ; 90.14] |  |  |
| Missing | 3 | 2 |  |  |
| **CD8+ / PD-1+ (Gated)** |  |  | 0.939^3^ | 0.993 |
| Mean (± SD) | 5.34 (± 4.11) | 5.10 (± 3.77) |  |  |
| Median (Q1 ; Q3) | 4.78 (1.66 ; 9.68) | 5.33 (1.77 ; 7.60) |  |  |
| [Min ; Max] | [0.18 ; 11.80] | [0.05 ; 13.47] |  |  |
| Missing | 3 | 2 |  |  |
| **CD-8+ / LAG-3+ (Gated)** |  |  | 0.188^2^ | 0.465 |
| Mean (± SD) | 74.60 (± 11.89) | 79.22 (± 10.95) |  |  |
| Median (Q1 ; Q3) | 76.93 (65.84 ; 81.82) | 79.51 (72.96 ; 88.82) |  |  |
| [Min ; Max] | [46.31 ; 91.00] | [56.45 ; 93.65] |  |  |
| Missing | 3 | 2 |  |  |
| **CD-8+ / TIGIT+ (Gated)** |  |  | 0.886^2^ | 0.993 |
| Mean (± SD) | 71.34 (± 24.38) | 72.35 (± 24.86) |  |  |
| Median (Q1 ; Q3) | 78.52 (53.05 ; 92.56) | 83.46 (53.99 ; 92.18) |  |  |
| [Min ; Max] | [23.24 ; 98.36] | [18.89 ; 98.06] |  |  |
| Missing | 3 | 2 |  |  |
| **CD-8+ / DNAM1+ (Gated)** |  |  | 0.846^2^ | 0.993 |
| Mean (± SD) | 90.97 (± 8.71) | 90.53 (± 9.00) |  |  |
| Median (Q1 ; Q3) | 94.04 (87.73 ; 97.66) | 94.06 (87.27 ; 96.42) |  |  |
| [Min ; Max] | [66.84 ; 98.72] | [68.43 ; 99.42] |  |  |
| Missing | 3 | 2 |  |  |
| **CD-8+ / NKG2A+ (Gated)** |  |  | 0.993^2^ | 0.993 |
| Mean (± SD) | 53.15 (± 23.03) | 53.81 (± 26.01) |  |  |
| Median (Q1 ; Q3) | 51.73 (38.48 ; 73.74) | 59.90 (32.68 ; 76.33) |  |  |
| [Min ; Max] | [13.96 ; 86.71] | [12.18 ; 91.65] |  |  |
| Missing | 3 | 2 |  |  |
| **CD3- / CD56+(Gated)** |  |  | 0.993^3^ | 0.993 |
| Mean (± SD) | 33.43 (± 16.82) | 33.30 (± 14.99) |  |  |
| Median (Q1 ; Q3) | 29.30 (21.99 ; 38.71) | 27.63 (23.97 ; 44.78) |  |  |
| [Min ; Max] | [6.84 ; 77.90] | [9.98 ; 68.16] |  |  |
| Missing | 3 | 2 |  |  |
| **CD56+ / CD4+ alone (Gated)** |  |  | 0.966^3^ | 0.993 |
| Mean (± SD) | 4.62 (± 3.89) | 4.21 (± 2.85) |  |  |
| Median (Q1 ; Q3) | 3.32 (1.97 ; 6.56) | 3.48 (2.09 ; 6.40) |  |  |
| [Min ; Max] | [0.24 ; 15.27] | [0.87 ; 10.55] |  |  |
| Missing | 3 | 2 |  |  |
| **CD56+ / CD8+ alone (Gated)** |  |  | 0.461^2^ | 0.856 |
| Mean (± SD) | 40.08 (± 13.12) | 37.20 (± 12.79) |  |  |
| Median (Q1 ; Q3) | 38.70 (34.10 ; 46.26) | 33.59 (27.99 ; 46.56) |  |  |
| [Min ; Max] | [11.56 ; 66.62] | [17.98 ; 63.71] |  |  |
| Missing | 3 | 2 |  |  |
| **CD56+ / TIGIT+ (Gated)** |  |  | 0.952^3^ | 0.993 |
| Mean (± SD) | 78.52 (± 23.13) | 77.09 (± 23.72) |  |  |
| Median (Q1 ; Q3) | 90.53 (62.02 ; 94.30) | 86.64 (56.06 ; 95.54) |  |  |
| [Min ; Max] | [32.03 ; 99.12] | [27.77 ; 99.03] |  |  |
| Missing | 3 | 2 |  |  |
| **CD56+ / DNAM1+ (Gated)** |  |  | 0.639^3^ | 0.993 |
| Mean (± SD) | 97.81 (± 2.53) | 98.09 (± 1.89) |  |  |
| Median (Q1 ; Q3) | 98.68 (97.67 ; 99.22) | 98.89 (96.95 ; 99.51) |  |  |
| [Min ; Max] | [90.03 ; 99.87] | [93.75 ; 99.97] |  |  |
| Missing | 3 | 2 |  |  |
| **CD11B+CD14+ / CD80-CD86- (Gated)** |  |  | 0.281^2^ | 0.561 |
| Mean (± SD) | 37.15 (± 18.82) | 31.05 (± 17.88) |  |  |
| Median (Q1 ; Q3) | 35.43 (20.56 ; 51.97) | 28.61 (19.48 ; 39.47) |  |  |
| [Min ; Max] | [8.70 ; 71.59] | [7.33 ; 76.86] |  |  |
| Missing | 3 | 1 |  |  |
| **CD11B+CD14+ / CD163-CD206+ (Gated)** |  |  | 0.993^2^ | 0.993 |
| Mean (± SD) | 15.11 (± 7.35) | 14.55 (± 6.86) |  |  |
| Median (Q1 ; Q3) | 15.05 (9.12 ; 18.73) | 16.15 (9.60 ; 20.11) |  |  |
| [Min ; Max] | [3.52 ; 41.29] | [1.65 ; 25.39] |  |  |
| Missing | 3 | 1 |  |  |
| **CD11B+CD14+ / CD163+CD206+ (Gated)** |  |  | 0.087^2^ | 0.322 |
| Mean (± SD) | 54.22 (± 23.15) | 44.15 (± 22.80) |  |  |
| Median (Q1 ; Q3) | 56.93 (33.95 ; 74.63) | 33.91 (25.16 ; 66.51) |  |  |
| [Min ; Max] | [13.41 ; 86.93] | [10.96 ; 89.77] |  |  |
| Missing | 3 | 1 |  |  |

| ^1^False discovery rate correction for multiple testing |
| --- |
| ^2^Wilcoxon rank sum exact test |
| ^3^Wilcoxon rank sum test |

Table S4: Comparison of plasma proteins in CTC-positive and CTC-negative patients with mBC (N=60: CTC>0 vs CTC=0)

| **Variable** | CTC>0  N = 35 | | | CTC=0  N = 25 | | **p-value** | | q-value1 | |
| --- | --- | --- | --- | --- | --- | --- | --- | --- | --- |
| **IL-8** |  | | |  | | **0.014^2^** | | 0.129 | |
| Mean (± SD) | 6.05 (± 1.53) | | | 5.07 (± 1.09) | |  | |  | |
| Median (Q1 ; Q3) | 6.02 (4.96 ; 6.65) | | | 4.98 (4.23 ; 5.53) | |  | |  | |
| [Min ; Max] | [3.74 ; 9.46] | | | [3.61 ; 8.26] | |  | |  | |
| Missing | 2 | | | 2 | |  | |  | |
| **IL-1 alpha** |  | | |  | | 0.233^2^ | | 0.526 | |
| Mean (± SD) | 0.74 (± 0.51) | | | 0.56 (± 0.46) | |  | |  | |
| Median (Q1 ; Q3) | 0.73 (0.51 ; 0.97) | | | 0.60 (0.48 ; 0.82) | |  | |  | |
| [Min ; Max] | [-0.69 ; 2.47] | | | [-0.78 ; 1.20] | |  | |  | |
| Missing | 2 | | | 2 | |  | |  | |
| **CD244** |  | | |  | | 0.201^2^ | | 0.476 | |
| Mean (± SD) | 5.90 (± 0.36) | | | 5.76 (± 0.30) | |  | |  | |
| Median (Q1 ; Q3) | 5.86 (5.69 ; 6.10) | | | 5.77 (5.60 ; 5.89) | |  | |  | |
| [Min ; Max] | [5.26 ; 6.93] | | | [5.13 ; 6.40] | |  | |  | |
| Missing | 2 | | | 2 | |  | |  | |
| **IL-6** |  | | |  | | 0.080^2^ | | 0.321 | |
| Mean (± SD) | 3.14 (± 1.12) | | | 2.72 (± 1.04) | |  | |  | |
| Median (Q1 ; Q3) | 3.01 (2.46 ; 3.72) | | | 2.19 (2.09 ; 3.15) | |  | |  | |
| [Min ; Max] | [1.24 ; 5.95] | | | [1.63 ; 6.03] | |  | |  | |
| Missing | 2 | | | 2 | |  | |  | |
| **ADGRG1** |  | | |  | | **0.043^2^** | | 0.225 | |
| Mean (± SD) | 1.87 (± 0.98) | | | 1.48 (± 0.85) | |  | |  | |
| Median (Q1 ; Q3) | 1.50 (1.16 ; 2.43) | | | 1.25 (0.93 ; 1.61) | |  | |  | |
| [Min ; Max] | [0.84 ; 4.72] | | | [0.40 ; 3.90] | |  | |  | |
| Missing | 2 | | | 2 | |  | |  | |
| **MUC-16** |  | | |  | | 0.173^2^ | | 0.459 | |
| Mean (± SD) | 4.66 (± 1.63) | | | 3.95 (± 1.45) | |  | |  | |
| Median (Q1 ; Q3) | 4.51 (3.12 ; 5.77) | | | 3.65 (2.82 ; 4.82) | |  | |  | |
| [Min ; Max] | [2.45 ; 8.07] | | | [1.54 ; 6.25] | |  | |  | |
| Missing | 2 | | | 2 | |  | |  | |
| **CD4** |  | | |  | | **0.015^2^** | | 0.129 | |
| Mean (± SD) | 3.28 (± 0.60) | | | 2.95 (± 0.31) | |  | |  | |
| Median (Q1 ; Q3) | 3.17 (2.96 ; 3.46) | | | 2.90 (2.72 ; 3.19) | |  | |  | |
| [Min ; Max] | [2.13 ; 5.27] | | | [2.32 ; 3.47] | |  | |  | |
| Missing | 2 | | | 2 | |  | |  | |
| **Galectin-9** |  | | |  | | **0.013^2^** | | 0.129 | |
| Mean (± SD) | 7.90 (± 0.44) | | | 7.66 (± 0.33) | |  | |  | |
| Median (Q1 ; Q3) | 7.83 (7.60 ; 8.14) | | | 7.59 (7.45 ; 7.83) | |  | |  | |
| [Min ; Max] | [7.05 ; 9.18] | | | [7.19 ; 8.78] | |  | |  | |
| Missing | 2 | | | 2 | |  | |  | |
| **CD40** |  | | |  | | **0.010^2^** | | 0.129 | |
| Mean (± SD) | 10.69 (± 0.73) | | | 10.30 (± 0.32) | |  | |  | |
| Median (Q1 ; Q3) | 10.59 (10.22 ; 10.94) | | | 10.27 (10.09 ; 10.47) | |  | |  | |
| [Min ; Max] | [9.67 ; 13.68] | | | [9.77 ; 11.03] | |  | |  | |
| Missing | 2 | | | 2 | |  | |  | |
| **CXCL1** |  | | |  | | 0.168^2^ | | 0.459 | |
| Mean (± SD) | 6.27 (± 1.12) | | | 5.88 (± 0.92) | |  | |  | |
| Median (Q1 ; Q3) | 6.12 (5.63 ; 7.00) | | | 5.77 (5.18 ; 6.42) | |  | |  | |
| [Min ; Max] | [4.30 ; 8.95] | | | [4.72 ; 8.12] | |  | |  | |
| Missing | 2 | | | 2 | |  | |  | |
| **TWEAK** |  | | |  | | 0.668^2^ | | 0.993 | |
| Mean (± SD) | 8.76 (± 0.53) | | | 8.80 (± 0.37) | |  | |  | |
| Median (Q1 ; Q3) | 8.79 (8.56 ; 8.95) | | | 8.80 (8.54 ; 9.06) | |  | |  | |
| [Min ; Max] | [7.10 ; 10.61] | | | [8.07 ; 9.62] | |  | |  | |
| Missing | 2 | | | 2 | |  | |  | |
| **FASLG** |  | | |  | | 0.608^2^ | | 0.993 | |
| Mean (± SD) | 7.35 (± 0.51) | | | 7.44 (± 0.51) | |  | |  | |
| Median (Q1 ; Q3) | 7.43 (7.07 ; 7.76) | | | 7.39 (7.16 ; 7.81) | |  | |  | |
| [Min ; Max] | [6.17 ; 8.13] | | | [6.43 ; 8.39] | |  | |  | |
| Missing | 2 | | | 2 | |  | |  | |
| **HGF** |  | | |  | | **0.008^2^** | | 0.129 | |
| Mean (± SD) | 9.61 (± 1.01) | | | 8.96 (± 0.49) | |  | |  | |
| Median (Q1 ; Q3) | 9.38 (8.95 ; 10.19) | | | 8.88 (8.65 ; 9.17) | |  | |  | |
| [Min ; Max] | [8.31 ; 12.91] | | | [8.17 ; 9.93] | |  | |  | |
| Missing | 2 | | | 2 | |  | |  | |
| **TNFRSF12A** |  | | |  | | **0.002^2^** | | 0.104 | |
| Mean (± SD) | 7.04 (± 0.92) | | | 6.45 (± 0.39) | |  | |  | |
| Median (Q1 ; Q3) | 6.81 (6.55 ; 7.37) | | | 6.38 (6.07 ; 6.67) | |  | |  | |
| [Min ; Max] | [5.80 ; 10.28] | | | [5.84 ; 7.29] | |  | |  | |
| Missing | 2 | | | 2 | |  | |  | |
| **CCL23** |  | | |  | | **0.025^2^** | | 0.184 | |
| Mean (± SD) | 11.74 (± 0.63) | | | 11.45 (± 0.38) | |  | |  | |
| Median (Q1 ; Q3) | 11.79 (11.44 ; 12.04) | | | 11.42 (11.17 ; 11.72) | |  | |  | |
| [Min ; Max] | [9.78 ; 12.84] | | | [10.87 ; 12.20] | |  | |  | |
| Missing | 2 | | | 2 | |  | |  | |
| **CCL3** |  | | |  | | **0.037^2^** | | 0.212 | |
| Mean (± SD) | 5.84 (± 0.68) | | | 5.43 (± 0.58) | |  | |  | |
| Median (Q1 ; Q3) | 5.73 (5.43 ; 6.16) | | | 5.28 (4.91 ; 5.83) | |  | |  | |
| [Min ; Max] | [4.31 ; 7.65] | | | [4.47 ; 6.60] | |  | |  | |
| Missing | 2 | | | 2 | |  | |  | |
| **PTN** |  | | |  | | 0.110^2^ | | 0.382 | |
| Mean (± SD) | 1.91 (± 0.91) | | | 1.62 (± 0.53) | |  | |  | |
| Median (Q1 ; Q3) | 1.90 (1.51 ; 2.48) | | | 1.64 (1.33 ; 1.82) | |  | |  | |
| [Min ; Max] | [0.04 ; 4.82] | | | [0.55 ; 2.99] | |  | |  | |
| Missing | 2 | | | 2 | |  | |  | |
| **MMP12** |  | | |  | | 0.139^2^ | | 0.426 | |
| Mean (± SD) | 7.33 (± 1.27) | | | 6.85 (± 0.88) | |  | |  | |
| Median (Q1 ; Q3) | 7.16 (6.57 ; 7.88) | | | 6.81 (6.50 ; 7.14) | |  | |  | |
| [Min ; Max] | [4.22 ; 10.19] | | | [4.94 ; 9.76] | |  | |  | |
| Missing | 2 | | | 2 | |  | |  | |
| **CXCL13** |  | | |  | | 0.069^2^ | | 0.321 | |
| Mean (± SD) | 8.76 (± 1.04) | | | 8.22 (± 0.72) | |  | |  | |
| Median (Q1 ; Q3) | 8.59 (8.02 ; 9.15) | | | 8.11 (7.56 ; 8.82) | |  | |  | |
| [Min ; Max] | [7.44 ; 11.46] | | | [7.24 ; 9.44] | |  | |  | |
| Missing | 2 | | | 2 | |  | |  | |
| **CSF-1** |  | | |  | | 0.077^2^ | | 0.321 | |
| Mean (± SD) | 9.28 (± 0.28) | | | 9.14 (± 0.27) | |  | |  | |
| Median (Q1 ; Q3) | 9.26 (9.13 ; 9.55) | | | 9.14 (8.92 ; 9.29) | |  | |  | |
| [Min ; Max] | [8.65 ; 9.75] | | | [8.69 ; 9.75] | |  | |  | |
| Missing | 2 | | | 2 | |  | |  | |
| ^1^False discovery rate correction for multiple testing | | | | | | | | | |
| ^2^Wilcoxon rank sum exact test | | | | | | | | | |
| ^3^Wilcoxon rank sum test  Table S5: Progression-free survival and clinical/biological characteristics (N=60 patients with mBC)   \| **Variable** \| **N** \| **HR^1^** \| **95% CI^1^** \| **P-value** \| \| --- \| --- \| --- \| --- \| --- \| \| **Age** \| 60 \| 0.98 \| 0.96, 1.01 \| 0.3 \| \| **Age** \| 60 \|  \|  \| 0.666 \| \| ≤60 years \|  \| — \| — \|  \| \| >60 years \|  \| 0.865 \| 0.44844, 1.66853 \|  \| \| **Neoadjuvant chemotherapy** \| 38 \|  \|  \| 0.193 \| \| No \|  \| — \| — \|  \| \| Yes \|  \| 1.74002 \| 0.77721, 3.89555 \|  \| \| **Radiotherapy** \| 38 \|  \|  \| 0.131 \| \| No \|  \| — \| — \|  \| \| Yes \|  \| 2.12564 \| 0.73264, 6.16720 \|  \| \| **Adjuvant chemotherapy** \| 38 \|  \|  \| 0.812 \| \| No \|  \| — \| — \|  \| \| Yes \|  \| 1.0964 \| 0.51388, 2.33928 \|  \| \| **Hormone therapy** \| 38 \|  \|  \| 0.277 \| \| No \|  \| — \| — \|  \| \| Yes \|  \| 1.85568 \| 0.55607, 6.19267 \|  \| \| **Synchronous metastasis** \| 60 \|  \|  \| **0.01** \| \| No \|  \| — \| — \|  \| \| Yes \|  \| 0.39213 \| 0.18280, 0.84116 \|  \| \| **Number of metastatic sites** \| 60 \| 1.21232 \| 0.93470, 1.57239 \| 0.171 \| \| **Number of metastatic sites** \| 60 \|  \|  \| 0.121 \| \| 1 \|  \| — \| — \|  \| \| ≥2 \|  \| 1.70231 \| 0.85713, 3.38089 \|  \| \| **Liver metastasis** \| 60 \|  \|  \| **0.004** \| \| No \|  \| — \| — \|  \| \| Yes \|  \| 2.86246 \| 1.44740, 5.66100 \|  \| \| **Bone metastasis** \| 60 \|  \|  \| 0.88 \| \| No \|  \| — \| — \|  \| \| Yes \|  \| 0.94873 \| 0.47965, 1.87655 \|  \| \| **Lung metastasis** \| 60 \|  \|  \| 0.665 \| \| No \|  \| — \| — \|  \| \| Yes \|  \| 1.17768 \| 0.56740, 2.44433 \|  \| \| **Histological type** \| 60 \|  \|  \| 0.531 \| \| Ductal invasive carcinoma \|  \| — \| — \|  \| \| Lobular carcinoma \|  \| 0.7457258 \| 0.2883834, 1.92836 \|  \| \| **SBR** \| 51 \|  \|  \| 0.153 \| \| 1 \|  \| — \| — \|  \| \| 2/3 \|  \| 2.21456 \| 0.66317, 7.39522 \|  \| \| **ER** \| 60 \|  \|  \| **0.032** \| \| Positive \|  \| — \| — \|  \| \| Negative \|  \| 3.01205 \| 1.22227, 7.42262 \|  \| \| **PR** \| 60 \|  \|  \| **0.01** \| \| Positive \|  \| — \| — \|  \| \| Negative \|  \| 2.45571 \| 1.24670, 4.83721 \|  \| \| **HR** \| 60 \|  \|  \| **0.043** \| \| Negative \|  \| — \| — \|  \| \| Positive \|  \| 0.32238 \| 0.12277, 0.84655 \|  \| \| **HER2** \| 60 \|  \|  \| 0.891 \| \| Positive \|  \| — \| — \|  \| \| Negative \|  \| 0.93547 \| 0.36147, 2.42094 \|  \| \| **Molecular subtypes** \| 60 \|  \|  \| 0.087 \| \| HR+/HER2- \|  \| — \| — \|  \| \| HR+/HER2+ \|  \| 0.67997 \| 0.16159, 2.86138 \|  \| \| HR-/HER2+ \|  \| 2.10637 \| 0.63286, 7.01066 \|  \| \| HR-/HER2- \|  \| 9.6599 \| 1.96781, 47.41993 \|  \| \| **CTC > 0** \| 60 \|  \|  \| **0.007** \| \| CTC = 0 \|  \| — \| — \|  \| \| CTC > 0 \|  \| 2.61897 \| 1.25743, 5.45481 \|  \| \| **CTC > 5** \| 60 \|  \|  \| **0.021** \| \| CTC < 5 \|  \| — \| — \|  \| \| CTC ≥ 5 \|  \| 2.20296 \| 1.14131, 4.25217 \|  \| \| ^1^HR = Hazard Ratio, CI = Exact Confidence Interval \| \| \| \| \|   Table S6: Progression-free survival and biological parameters | | | | | | | | | |
| Variable | | N | HR^1^ | | 95% CI^1^ | | P-value | |  |
| % Gated CD3^+^/PD-1^+^ | | 55 | 1.05478 | | 0.96951, 1.14754 | | 0.223 | |  |
| % Gated CD3^+^/CTLA-4^+^ | | 55 | 0.99750 | | 0.97995, 1.01537 | | 0.780 | |  |
| % Gated CD3^+^/LAG-3^+^ | | 55 | 1.00525 | | 0.98022, 1.03092 | | 0.682 | |  |
| % Gated CD3^+^/TIGIT^+^ | | 55 | 0.99952 | | 0.98787, 1.01131 | | 0.936 | |  |
| % Gated CD4^+^/Treg^+^ | | 55 | 1.04359 | | 0.84472, 1.28927 | | 0.694 | |  |
| % Gated CD4^+^/PD-1^+^ | | 55 | 1.05092 | | 0.96979, 1.13883 | | 0.236 | |  |
| % Gated CD4^+^/CTLA-4^+^ | | 55 | 0.99632 | | 0.97844, 1.01452 | | 0.684 | |  |
| % Gated CD4^+^/LAG-3^+^ | | 55 | 1.00399 | | 0.98074, 1.02779 | | 0.738 | |  |
| % Gated CD4^+^/TIGIT^+^ | | 55 | 0.99885 | | 0.98782, 1.01000 | | 0.839 | |  |
| % Gated Treg^+^/PD-1^+^ | | 55 | 1.03347 | | 0.95849, 1.11431 | | 0.402 | |  |
| % Gated Treg^+^/CTLA-4^+^ | | 55 | 0.99699 | | 0.98060, 1.01366 | | 0.718 | |  |
| % Gated Treg^+^/LAG-3^+^ | | 55 | 0.99816 | | 0.97399, 1.02293 | | 0.883 | |  |
| % Gated Treg^+^/TIGIT^+^ | | 55 | 0.99891 | | 0.98580, 1.01220 | | 0.872 | |  |
| % Gated CD8^+^/PD-1^+^ | | 55 | 1.03340 | | 0.94100, 1.13489 | | 0.495 | |  |
| % Gated CD8^+^/CTLA-4^+^ | | 55 | 0.99578 | | 0.97769, 1.01421 | | 0.646 | |  |
| % Gated CD8^+^/LAG-3^+^ | | 55 | 1.00160 | | 0.97299, 1.03106 | | 0.914 | |  |
| % Gated CD8^+^/TIGIT^+^ | | 55 | 0.99973 | | 0.98577, 1.01389 | | 0.970 | |  |
| % Gated CD3^-^/CD19^+^ | | 55 | 1.00500 | | 0.97836, 1.03237 | | 0.715 | |  |
| % Gated CD3^-^/CD56^+^ | | 55 | 0.99001 | | 0.96445, 1.01624 | | 0.444 | |  |
| % Gated CD56^+^/TIGIT^+^ | | 55 | 0.99774 | | 0.98317, 1.01252 | | 0.764 | |  |
| % Gated CD11B^+^CD14^+^/ CD80^+^CD86^+^ | | 56 | 1.01692 | | 0.99314, 1.04126 | | 0.182 | |  |
| % Gated CD11B^+^CD14^+^/ CD163+CD206+ | | 56 | 0.99967 | | 0.98449, 1.01508 | | 0.966 | |  |
| IL-8 | | 56 | 1.61508 | | 1.26935, 2.05497 | | <0.001 | |  |
| IL-1 alpha | | 56 | 1.68692 | | 0.85711, 3.32011 | | 0.134 | |  |
| CD244 | | 56 | 0.99701 | | 0.36754, 2.70455 | | 0.995 | |  |
| EGF | | 56 | 1.02330 | | 0.79143, 1.32311 | | 0.860 | |  |
| IL-6 | | 56 | 1.25813 | | 0.93058, 1.70098 | | 0.154 | |  |
| ADGRG1 | | 56 | 1.59057 | | 1.13461, 2.22976 | | 0.012 | |  |
| CXCL9 | | 56 | 1.19211 | | 0.86435, 1.64417 | | 0.299 | |  |
| MUC-16 | | 56 | 1.19257 | | 0.94973, 1.49750 | | 0.133 | |  |
| CD4 | | 56 | 1.30940 | | 0.74329, 2.30669 | | 0.369 | |  |
| NOS3 | | 56 | 6.66065 | | 2.54981, 17.39906 | | <0.001 | |  |
| Galectin-9 | | 56 | 1.83341 | | 0.81696, 4.11451 | | 0.157 | |  |
| CD40 | | 56 | 1.59287 | | 1.06719, 2.37749 | | 0.050 | |  |
| CXCL1 | | 56 | 1.35890 | | 1.00682, 1.83411 | | 0.051 | |  |
| TWEAK | | 56 | 0.32764 | | 0.13132, 0.81744 | | 0.022 | |  |
| FASLG | | 56 | 0.44297 | | 0.21683, 0.90495 | | 0.026 | |  |
| CCL19 | | 56 | 0.95007 | | 0.59904, 1.50680 | | 0.827 | |  |
| HGF | | 56 | 2.28911 | | 1.41751, 3.69663 | | 0.001 | |  |
| TNFRSF12A | | 56 | 1.68250 | | 1.23954, 2.28376 | | 0.005 | |  |
| CCL23 | | 56 | 0.98905 | | 0.48546, 2.01503 | | 0.976 | |  |
| ANGPT2 | | 56 | 2.60573 | | 1.45744, 4.65873 | | 0.001 | |  |
| PTN | | 56 | 1.60170 | | 0.90445, 2.83648 | | 0.100 | |  |
| MMP12 | | 56 | 1.27233 | | 0.92268, 1.75449 | | 0.159 | |  |
| CXCL13 | | 56 | 1.60280 | | 1.09335, 2.34963 | | 0.021 | |  |
| CSF-1 | | 56 | 4.32599 | | 1.27388, 14.69078 | | 0.019 | |  |
| CCL3 | | 56 | 1.53017 | | 0.91305, 2.56438 | | 0.116 | |  |
| ^1^HR = Hazard Ratio, CI = Exact Confidence Interval | | | | | | | | |  |
